# Selective Requirements for Vascular Endothelial Cells and Circulating Factors in the Regulation of Retinal Neurogenesis

**DOI:** 10.3389/fcell.2021.628737

**Published:** 2021-04-08

**Authors:** Susov Dhakal, Shahar Rotem-Bamberger, Josilyn R. Sejd, Meyrav Sebbagh, Nathan Ronin, Ruth A. Frey, Mya Beitsch, Megan Batty, Kineret Taler, Jennifer F. Blackerby, Adi Inbal, Deborah L. Stenkamp

**Affiliations:** ^1^Department of Biological Sciences, University of Idaho, Moscow, ID, United States; ^2^Department of Medical Neurobiology, Institute for Medical Research Israel-Canada, The Hebrew University-Hadassah Medical School, Jerusalem, Israel; ^3^Department of Biology, Gonzaga University, Spokane, WA, United States

**Keywords:** retina, vascular endothelial cell, neurogenesis, differentiation, circulation, zebrafish, development, eye

## Abstract

Development of the vertebrate eye requires signaling interactions between neural and non-neural tissues. Interactions between components of the vascular system and the developing neural retina have been difficult to decipher, however, due to the challenges of untangling these interactions from the roles of the vasculature in gas exchange. Here we use the embryonic zebrafish, which is not yet reliant upon hemoglobin-mediated oxygen transport, together with genetic strategies for (1) temporally-selective depletion of vascular endothelial cells, (2) elimination of blood flow through the circulation, and (3) elimination of cells of the erythroid lineage, including erythrocytes. The retinal phenotypes in these genetic systems were not identical, with endothelial cell-depleted retinas displaying laminar disorganization, cell death, reduced proliferation, and reduced cell differentiation. In contrast, the lack of blood flow resulted in a milder retinal phenotype showing reduced proliferation and reduced cell differentiation, indicating that an endothelial cell-derived factor(s) is/are required for laminar organization and cell survival. The lack of erythrocytes did not result in an obvious retinal phenotype, confirming that defects in retinal development that result from vascular manipulations are not due to poor gas exchange. These findings underscore the importance of the cardiovascular system supporting and controlling retinal development in ways other than supplying oxygen. In addition, these findings identify a key developmental window for these interactions and point to distinct functions for vascular endothelial cells vs. circulating factors.

## Introduction

The vertebrate eye develops as an intricate collaboration among multiple tissue types (Bryan et al., 2020). These tissues are derived from the neuroepithelium of the neural tube, mesenchyme of mesodermal and neural crest origin, and surface ectoderm. Numerous developmental signals and cell surface interactions among these tissues are required for specification, patterning, differentiation, and growth of each structure and specialized tissue of the eye. For example, in mouse, Wnt signals from extraocular mesenchyme are needed to pattern and maintain the posterior regions of the developing optic vesicle as retinal pigmented epithelium (RPE) rather than as neural retina (Bankhead et al., 2015). As another example, an unknown factor from the lens is required for maturation of the hyaloid vasculature in zebrafish (Hartsock et al., 2014).

In the present study we focus upon furthering our understanding of developmental signals derived from the vasculature, which may regulate or support the development of the neural retina. Developmental roles for the vascular endothelial cells in the genesis and differentiation of neurons and glia of the CNS have predominantly been addressed in previous studies using cell culture models, in the context of understanding neural stem and progenitor cell niches. In three-dimensional co-culture of endothelial cells and retinal progenitor cells, the presence of endothelial cells inhibited proliferation and differentiation of the retinal cells (Aizawa and Shoichet, 2012). In contrast, an alternative *in vitro* approach suggested that soluble factors from endothelial cells promote neural stem cell self-renewal while inhibiting their differentiation (Shen et al., 2004). The understanding of these interactions *in vivo* is even less clear, due to the challenges in uncoupling developmental functions of the vasculature from its roles in gas exchange and nutrient delivery. Gain-of-function approaches (increasing vessel density or size), however, have been informative. In mouse models in which supernumerary blood vessels form, retinal dysplasias resulted (Rutland et al., 2007), specifically retinal folds, retinal detachment, and disrupted retinal lamination (Zhang et al., 2008). In a zebrafish model displaying an enlarged hyaloid vein, the optic fissure fails to close (Weiss et al., 2012). The invasion and elaboration of blood vessels of the eye correspond temporally with critical developmental events of retinal morphogenesis, proliferation, and differentiation in vertebrates (Hu and Easter, 1999; Baba et al., 2009; Graw, 2010; McLeod et al., 2012; Hartsock et al., 2014; Kaufman et al., 2015). Therefore, it is important to further understand specific developmental functions of the vasculature, using loss-of-function approaches. An increased understanding of these functions would also have applications for the many retinal disorders that involve abnormalities of the vasculature, including age-related macular degeneration (van Lookeren Campagne et al., 2014), retinopathy of prematurity (Hartnett, 2015), and diabetic retinopathy (Shin et al., 2014).

Formation of ocular vasculature in the zebrafish embryo temporally correlates with retinal neurogenesis. The earliest component of this vascular system is the hyaloid artery, that grows into the eye around 18-20 h post-fertilization (hpf) and subsequently develops into a network of hyaloid vessels that cradles the lens (Hartsock et al., 2014). Another set of blood vessels is the superficial system that begins to develop around 24 hpf and is completed by approximately 54 hpf. The completed system appears as a ring-like structure surrounding the lens and is supplied by a nasal radial vessel (nrv), through which blood enters the system, along with dorsal and ventral radial vessels (drv, vrv), through which blood is drained out of the eye (Kitambi et al., 2009; Kaufman et al., 2015). Over the same time period, soon after 24 hpf and until 72 hpf, retinal progenitor cells differentiate into neurons and glia of the retina. Beginning at 28 hpf, retinal ganglion cells differentiate, followed by neurons located in the inner nuclear layer and then by photoreceptors, such that by 72 hpf the retina is differentiated and functional [reviewed in (Stenkamp, 2015)]. This correlation in the timing of development of retina and its vasculature raises the question of how development of one of these tissues might affect the other.

In our previous study (Dhakal et al., 2015), we made an important step toward learning specific developmental functions of eye vasculature for retinal development. We took advantage of the ability of the tissues of the zebrafish embryo to achieve adequate gas exchange by diffusion (Pelster and Burggren, 1996), and the availability of the *cloche/npas4l* mutant. *cloche* mutants lack most blood cells, endothelial cells, and endocardial cells (Stainier et al., 1995) due to a mutation in *npas4l* (Reischauer et al., 2016; Marass et al., 2019), and are a useful model for understanding roles of the vasculature for organ development that are not strictly metabolic (Takada et al., 2017). We found that the retinas of *cloche−/−* embryos displayed defects in laminar organization, cell proliferation, cell survival, and cell differentiation, without molecular evidence of hypoxia at the age of analysis (Dhakal et al., 2015). However, the functions of vascular endothelial cells vs. functions of circulating factors or specific blood cell types remain to be determined, since in the previous study, all of these were disrupted. In addition, the developmental timeframe over which the vasculature was required for the discovered functions was not investigated.

In the present study we again use the informative embryonic zebrafish model, but with strategies that more selectively target components of the vascular system, to unravel the contributions of endothelial cells vs. circulating factors for supporting and regulating retinal development. We find that the presence of endothelial cells is needed for retinal organization and retinal cell survival, while the presence of circulation – likely contributing circulating factors – also participates in the control of retinal cell proliferation and retinal cell differentiation. One of our strategies specifically disrupted the vasculature from 48 hpf to 72 hpf, a period of expansion of the ocular vasculature (Hartsock et al., 2014), and retinal differentiation (Raymond et al., 1995; Hu and Easter, 1999). This temporally-selective ablation of vessels produced a near-phenocopy of the *cloche* mutant retinal phenotype, suggesting this developmental window is a key period of vessel-retina interactions.

## Materials and Methods

### Animals

Adult zebrafish were maintained as described (Westerfield, 2007) at 28.5°C in aquatic housing units with recirculating water and in a 14-h light cycle. The embryo was considered 0 h post-fertilization (hpf) at the time of spawning, and the embryos were staged as described (Westerfield, 2007). For some experiments, embryos were treated with 0.003% phenylthiourea (PTU) to inhibit melanin synthesis (Westerfield, 2007). PTU treatment facilitated visualization of fluorescence transgenic reporters and the measurement of lens size. When large clutches were obtained, PTU was also administered so that embryos could be used for more than one type of analysis in parallel. *tnnt2a*^*mn0031Gt*^ (a.k.a., *silent heart* or *sih*) zebrafish were the kind gift of Jeff Essner. In this line, a gene-breaking transposon disrupts the *tnnt2a* (encoding cardiac troponin T2a) locus with a floxed RFP, resulting in loss-of-function of cardiac contractility in homozygous transgenics, and fluorescent hearts in homozygous transgenics and in hemizygotes (Clark et al., 2011). *gata1a^*m651*^ (a.k.a., vlad tepes* or *vlt*) carriers were obtained from the Zebrafish International Resource Center (ZIRC), and homozygous mutants were identified by evaluation for the presence vs. absence of circulating blood cells under a dissecting microscope (Lyons et al., 2002). Some of the carriers of *sih* or *vlt* mutations were maintained on transgenic backgrounds permitting visualization of blood vessels and/or blood cells, either *Tg(kdrl:eGFP)s843* (Jin et al., 2005), the gift of Didier Stainier, or *Tg(gata1:dsRed)sd2* (ZIRC). *Tg(cdh5:GAL4ff)mu101* was the generous gift of Jesus Torres Vazquez (Bussmann et al., 2011). *Tg(UAS-E1b:Eco.NfsB-mCherry)c264* was purchased from ZIRC. The Institutional Animal Care and Use Committees (IACUC) of University of Idaho and of Hebrew University of Jerusalem approved the animal protocols used in this study.

### Experimental Depletion of Endothelial Cells

Doubly transgenic zebrafish embryos were obtained from crossing homozygous (or hemizygous) *Tg(cdh5:GAL4ff)* with hemizygous *Tg(UAS-E1b:Eco.NfsB-mCherry).* Half of the embryos within each clutch were treated with 10mM metronidazole (Met) (Sigma-Aldrich) prepared in 0.2% dimethylsulfoxide (DMSO), beginning at 12 hpf, and were maintained in the dark thereafter to prevent photo-inactivation (Curado et al., 2008). Met solution was replaced at 24 hpf and after every exposure to light (e.g., sorting/identification of doubly transgenic embryos). The other half of the embryos were treated with the 0.2% DMSO vehicle, and also maintained in the dark. The doubly transgenic embryos in the Met group and DMSO group were identified at 30 hpf, based upon mCherry expression in vascular endothelial cells. Other embryos within each group were considered to be either singly transgenic or wild type (WT). Therefore, each experiment included Met-treated, doubly transgenic embryos, Met-treated, single transgenics or WT embryos (“Met controls”), and DMSO-treated, doubly transgenic embryos (DMSO controls).

**FIGURE 1:**
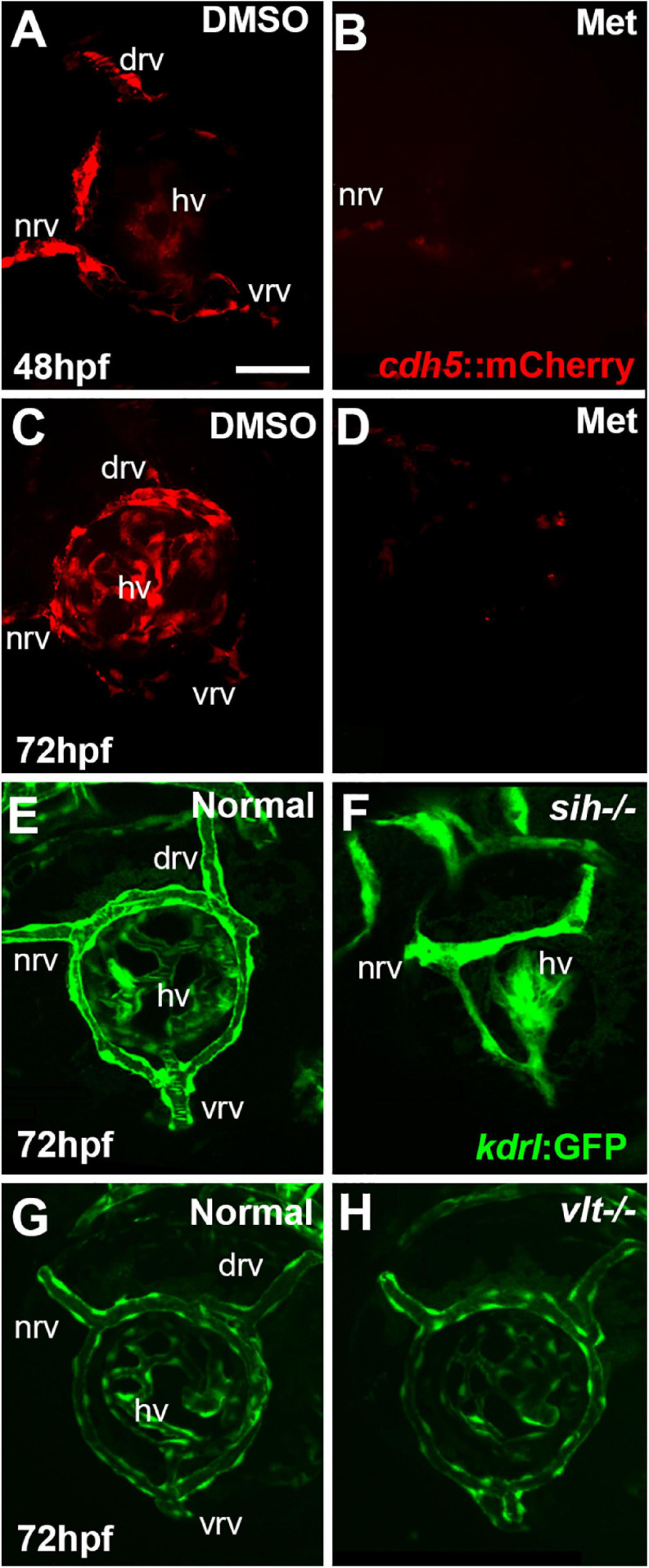
Ocular vasculature of cardiovascular disruption model systems. **(A,D)**. Hyaloid and superficial vessels in zebrafish embryos doubly transgenic for *cdh5:gal4* and *UAS:nfsB-mCherry*, treated with DMSO (controls; **A,C)**, or metronidazole (Met; **B,D)**, viewed at 48 h post-fertilization (hpf; **A,B)** and 72 hpf **(C,D)**. The dorsal, nasal, and ventral radial vessels (drv, nrv, vrv, respectively) and hyaloid vasculature (hv) are visible in controls but not in Met-treated. **(E,F)** Hyaloid and superficial vessels of *kdrl:eGFP* transgenic zebrafish embryos, either normal **(E)** or *sih–/–* with no circulation and collapsed vessels **(F)**. **(G,H)** Hyaloid and radial vessels of *kdrl:eGFP* transgenic zebrafish embryos, either normal **(G)** or *vlt–/–* with no erythrocytes but otherwise normal vasculature **(H)**. Scale bar in A (applies to all) = 50 μm. 7-10 embryos were examined for each condition. All embryos were PTU-treated to prevent pigmentation from interfering with imaging.

**FIGURE 2:**
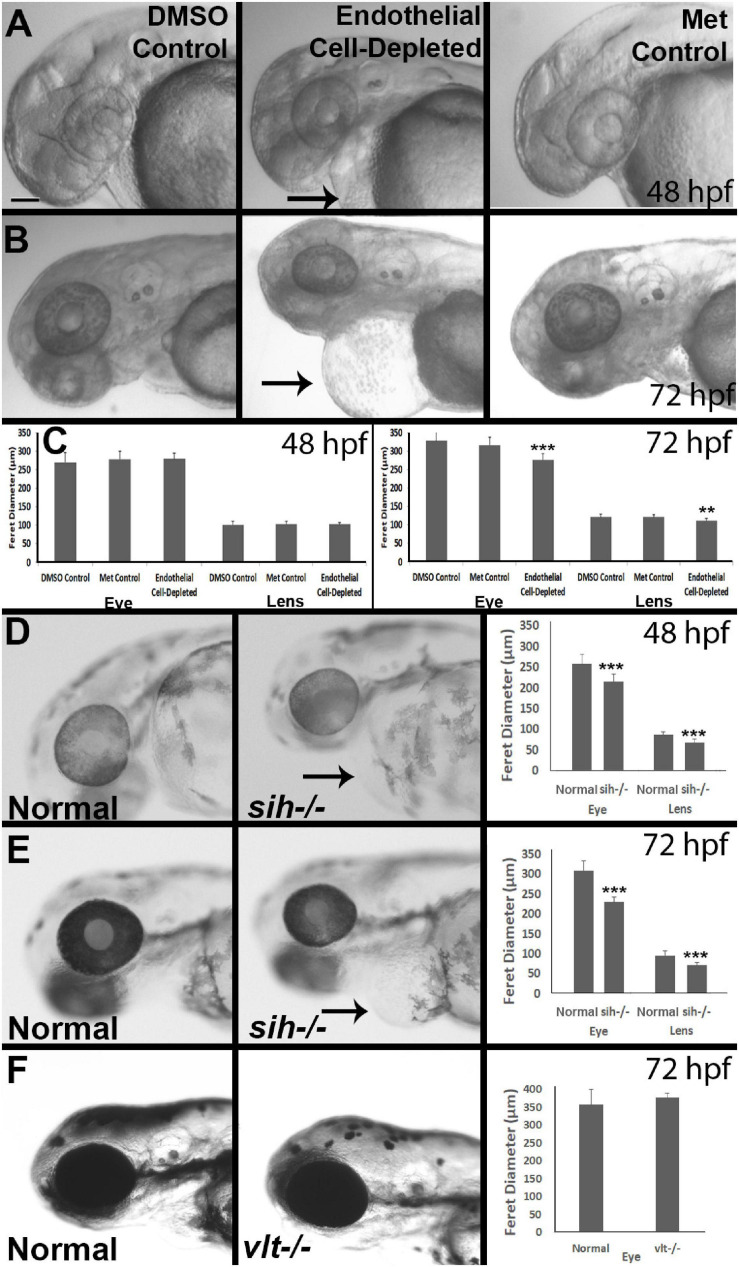
Eye and lens sizes in cardiovascular disruption model systems. **(A,B)** Live, doubly-transgenic (*cdh5:gal4; UAS:nfsB-mCherry*), DMSO-treated (DMSO Control), Met-treated (Endothelial Cell-Depleted), and Met-treated clutchmates (Met controls) viewed at 48 hpf **(A)** and 72 hpf **(B)**. Arrows in middle panel of each indicate swollen epicardial sac. **(C)**. Graphs show average (±s.d.) Feret diameter of eyes and lenses; statistical analysis by ANOVA and Tukey *post hoc* (48 hpf; *n* = 15 per condition) and Kruskal-Wallis and Conover’s *post hoc* (72 hpf; *n* = 15 DMSO, 14 Met control, and 12 Endothelial cell-depleted). **(D,E)** Live, normal clutchmates and *sih–/–* embryos viewed at 48 hpf **(D)** and 72 hpf **(E)**. Arrows in second panel of each indicate swollen epicardial sac. Graphs in **(D,E)** show average (±s.d.) Feret diameter of eyes and lenses; statistical analyses by Student’s *t*-tests (48 hpf; *n* = 22 normal, 7 *sih–/–*) and Mann-Whitney test (72 hpf; *n* = 17 normal, 11 *sih–/–*). **(F)** Live, normal clutchmates and *vlt–/–* embryos viewed at 72 hpf. Graph in **(F)** shows average (±s.d.) Feret diameter of eyes; statistical analysis by Mann-Whitney test (*p* = 0.068; *n* = 12 normal, 13 *vlt–/–*). Scale bar (in **(A)**, applies to all images) = 100 μm. Statistical notation: **p < 0.01; ***p < 0.001. Embryos in **(A–E)** were PTU-treated to prevent pigmentation from interfering with visualizing the lens.

We verified Met-mediated depletion of vascular endothelial cells in several ways. Firstly, we established a timeline by which substantial loss of ocular endothelial cells was achieved by Met treatment in doubly transgenic embryos. Substantial loss of these cells was presumed to occur when there was no visible blood circulation present in the embryos despite the presence of heart beat. We viewed the embryos under a dissecting microscope at selected developmental time points and tracked the number of embryos with vs. without circulating materials. Secondly, we used a Nikon-Andor spinning disk or Zeiss LSM 700 confocal microscope to visualize mCherry+ ocular vessels at 48 and 72 hpf. Zebrafish embryos were fixed using 4% paraformaldehyde for 1 h at room temperature followed by three, 30 min rinses in PBS. The embryos were then stored in PBS at 4°C. Before imaging, the fixed whole embryos were mounted in 0.1% low melting point agarose. The images were collected at 0.4-μm steps for at least 45 optical sections per eye using an Apo LWD 40X lens with a 561nm solid-state laser. The images were processed using NIS elements viewer software (Figure 1A). Thirdly, because the mCherry reporter did not always report all endothelial cells in control embryos (Figure 1A), we crossed these lines onto *kdrl:GFP* and *gata1:dsRed* backgrounds to better visualize vessels and blood cells in controls, and their absence in doubly-transgenic Met-treated embryos (Supplementary Figure 1). The treated embryos were mounted in 0.5% low melting point agarose (Lonza, 50101) in the presence of 0.01% tricaine (MS-222; Sigma) to block movement and were subjected to live imaging as previously described (Kaufman et al., 2015). Fourthly, we verified vasculature reduction by imaging eyes of living embryos for the presence of circulating materials through the ocular vessels, at 48 hpf. Live embryos were immobilized using tricaine, followed by mounting the whole embryos in 0.1% low melting point agarose prepared in tricaine solution, with an eye facing the objective. The eyes were imaged in a single focal plane continuously with an Apo LWD 40X lens for 1 min with each image exposed for 40 msec under transmitted light to visualize circulating materials in the hyaloid vasculature (Movies 1-3). Finally, the presence of a swollen epicardial sac was evident at 48 hpf and beyond in embryos lacking circulation (Figure 2A). This heart abnormality likely arose due to the destruction of endocardial cells, which, like endothelial cells, also express *cdh5* (Larson et al., 2004).

The condition of the hyaloid and superficial retinal vessels of *sih* and *vlt* mutants was assessed by confocal imaging of embryos carrying the *kdrl:eGFP* transgene (Figure 1).

### Eye Measurements

In most cases, embryos were treated with 0.003% phenylthiourea (PTU) to inhibit melanin synthesis (Westerfield, 2007) to facilitate visualization of eyes and the lenses within the eyes. To directly measure eye and lens size, live, anaesthetized embryos were positioned with their sides facing up on a petri dish and imaged with a Nikon stereomicroscope with a CCD camera (Meridian Instruments, Freeland, WA), or were mounted in 2% methyl cellulose and imaged with a Zeiss Discovery.V8 stereomicroscope equipped with an Axiocam MRc digital camera. The images were collected such that clear boundaries of the eye and the lens were in same focal plane. Perimeters were drawn around the eyes and lenses within each image, and Feret’s diameter (estimates diameter of irregularly shaped objects) was measured for each using FIJI (ImageJ) software.

### Tissue Preparation: Plastic Sections

Embryos were fixed overnight in 4% paraformaldehyde at 4°C, washed with phosphate-buffered saline containing 0.1% Tween-20 (J.T.Baker) (PBT), dehydrated in an ethanol series, and then embedded in JB4 resin (Polysciences, Inc.), using the manufacturer’s instructions. An LKB 8800 Ultratome II microtome was used to cut 4 μm sections. Sections were stained with methylene blue-azure II (Humphrey and Pittman, 1974) and mounted in Permount (Fisher Chemical).

### Tissue Preparation: Cryosections

Embryos were fixed in 4% paraformaldehyde in 5% sucrose, phosphate-buffered (pH = 7.4) solution, or in phosphate-buffered (pH = 7.4) saline containing 0.1% Tween-20 (PBT) for 1 h at room temperature (Dhakal et al., 2015). Fixed embryos were washed by incubating them in increasing concentrations of sucrose and were cryoprotected overnight with gentle agitation at 4°C in phosphate-buffered 20% sucrose solution. The fixed embryos were embedded in one part OCT medium (Sakura Finetek, Torrance, CA), two parts phosphate-buffered 20% sucrose, frozen using 2-methylbutane on liquid nitrogen, and the tissue blocks were stored at −20°C. Alternatively, embryos were embedded in 1.2% agarose + 5% sucrose in water. Agarose blocks were kept in 30% sucrose overnight at 4°C and then frozen using 2-methylbutane on liquid nitrogen. Sections (5-16 μm) were collected using a Leica CM3050 cryostat. The slides were dried in a vacuum desiccator overnight and stored at −20°C.

### Hematoxylin and Eosin

Retinal lamination was also analyzed using a hematoxylin (Sigma-Aldrich) and eosin (Sigma-Aldrich) staining procedure. Briefly, the slides were hydrated in PBS followed by incubation in hematoxylin for 1-5 min followed by a tap water rinse. Then, the slides were incubated in eosin for 1-2 min followed by rinsing with distilled water. Finally, the slides were dehydrated in the increasing concentrations of EtOH and xylene and mounted in Permount (Fisher Scientific).

### Immunofluorescence and *in situ* Hybridization

Indirect immunofluorescence experiments were performed as previously described (Stevens et al., 2011; Dhakal et al., 2015). Briefly, prior to addition of primary antibodies, the slides were incubated with a blocking solution consisting of 20% goat serum in PBST for 30 min. The following primary antibodies were used: mouse monoclonal Zn8, labels retinal ganglion cells and their axons (Hu and Easter, 1999) (1:10, ZIRC); mouse monoclonal 1D1, labels Rhodopsin (Fadool, 2003) (1:100, generous gift from James Fadool); mouse monoclonal zpr1, targets Arrestin3a (Renninger et al., 2011) in red and green-sensitive double cones (Larison and Bremiller, 1990) (1:100, ZIRC); mouse monoclonal zrf1, labels Glial fibrillary acidic protein (GFAP) in Müller glia (Marcus and Easter, 1995) (1:400, ZIRC); mouse monoclonal anti Glutamine synthetase (GS), labels Müller glia (Peterson et al., 2001) (1:500, BD Biosciences); mouse monoclonal SV2, labels Synaptic vesicle 2 (1:2000, Developmental Studies Hybridoma Bank); rabbit polyclonal cleaved caspase-3, labels apoptotic cells (1:200, AbCam); rabbit polyclonal antiphosphohistone-3, labels cells in M-phase (PH3, 1:1000, Cell Signaling Technologies); rabbit anti L-plastin, labels pan-leucocytes including microglia (Le Guyader et al., 2008; Mitchell et al., 2018; Blume et al., 2020) (1:10000, generous gift of Michael Redd). Tissue sections were incubated at 4°C overnight, in primary antibody suspended in PBST containing 1% goat serum. Primary antibody was detected using either fluorescein conjugated secondary antibody (1:200 or 1:500) or Cy3-conjugated secondary antibody (1:200 or 1:500). In some cases, the sections were counterstained with 0.5-1.0 μg/mL 4′,6-diaminidino-2-phenylindole (DAPI), included in the secondary antibody application. The slides were mounted with Vectashield mounting medium (Vector labs).

*In situ* hybridizations were performed as previously described (Mitchell et al., 2015; Mackin et al., 2019). *In vitro* transcription was used to generate digoxigenin labeled cRNA probe from the plasmids. *pax6a* and *crx* cDNAs were the gifts of Pamela Raymond; *neuroD1* cDNA was the gift of Peter Hitchcock.

### Photography

For epifluorescence and differential interference contrast (DIC/Nomarski) images of sections, a Leica DMR compound microscope with SPOT camera system (Diagnostic Instruments), or a Leica DM2500 compound microscope equipped with a Leica DFC7000T camera was used. Alternatively, an Axio Imager.M2 compound microscope with MRc Digital camera was used. Figures were assembled using Adobe PhotoShop CS6.

### Cell Quantification

The following methods were used to count the fluorescently-labeled cells. *Rod photoreceptors:* All 1D1-positive rod photoreceptors located dorsal to the optic nerve were counted to determine the average number of 1D1+ cells per section per retina. The dense patch of rods located in the ventral retina was excluded from this quantification because the density of rods in this region precludes accurate counting (Raymond et al., 1995; Stevens et al., 2011). *Cone photoreceptors:* Zpr1+ cone labeling was scored using three categories (*None* = no labeled cells; *Few* = 1 to 10 labeled cells; *Many* > 10 labeled cells) (Kashyap et al., 2011; Nelson et al., 2009; Dhakal et al., 2015). *Müller glia:* GS+ cell bodies within the INL and displaying Müller glia morphology were counted to estimate the average number of Müller glia present per section per retina. *Microglia:* Microglial cells labeled by L-plastin antibody were counted to obtain average number of microglia present per section per retina. *Cell proliferation:* Nuclei of proliferating cells in M-phase labeled by PH3 antibody were counted to estimate the average number of proliferating cells per section per retina. *Cell death:* Cells undergoing apoptosis were labeled by anti-CC3 antibody. Total numbers of CC3+ profiles in the retina were counted to determine average number of CC3+ cells per section per retina. CC3+ cells that were at least 3 microns in diameter were only included in the count to maintain consistency and to avoid counting cell fragments. *The (zn8+) ganglion cell layer (GCL) and the (SV2+) inner plexiform layer (IPL):* Within images of fluorescently-labeled cryosections, perimeters were drawn around the zn8+ GCL and the SV2+ IPL, respectively, using the freehand selection tool in FIJI. A perimeter was also drawn around the retina (excluding lens and RPE) and ratios of GCL/retina and IPL/retina were calculated. In all cases of cell or layer quantification, to avoid double counting, only those sections that were at least 15 microns apart were used.

### Analysis of Hypoxia by Quantitative Reverse Transcriptase Polymerase Chain Reaction (qPCR) Analysis of *phd3/egln3*

Hypoxic samples of wild-type embryos were prepared as described (Tucker et al., 2011; Dhakal et al., 2015). In brief, filtered system water was boiled to remove dissolved gasses. 0.2 mg/mL sodium sulfite was added, and then this water was cooled to 28.5°C under N_2_ gas. O_2_ levels, measured with a YSI DO200 O_2_ meter, were 0.08-0.12 ppm. Embryos were incubated in this deoxygenated water for 90 min in airtight, 250 mL flasks, and then transferred to individual culture wells containing normoxic system water for 30 min prior to RNA extraction.

Whole embryos were submerged in RNALater (Life Technologies) and then stored at −80°C. The High Capacity cDNA Reverse Transcription kit (Applied Biosystems; ABI, Foster City, CA) was used to synthesize cDNA with random primers, from total RNA. qPCR amplification was performed with a model 7900HT fast Real-Time PCR System, and SYBR-Green PCR Master Mix (ABI). Primer pairs targeting *phd3* were 5′ CGCTGCGTCACCTGTATT 3′ (forward) and 5′ TAGCATACGACGGCTGAACT 3′ (reverse) (Santhakumar et al., 2012). Primer pairs targeting 18s were 5′ GAACGCCACTTGTCCCTCTA 3′ (forward) and 5′ GTTGGTGGAGCGATTTGTCT 3′ (reverse) (Sherpa et al., 2014). Transcript abundance was quantified as relative levels between control and experimental treatments using the ddCT method, with 18s ribosomal RNA as the endogenous reference.

### Statistics

Quantitative, parametric data describing each experimental condition within an analysis were subjected to a Shapiro-Wilk test for a normal distribution. If, for all conditions of an analysis the null hypothesis could be accepted (that the data displayed normal distributions), then statistical comparisons were made using either Student’s *t*-test (comparison of two conditions), or ANOVA with Tukey’s *post hoc* analysis (comparisons involving three conditions). If, for any condition of an analysis the null hypothesis was rejected, then statistical comparisons were made using either a Mann-Whitney *U* test (comparison of two conditions), or a Kruskal-Wallis test followed by a Conover *post hoc* analysis (comparisons involving three conditions). Non-parametric data (e.g., ratios) were analyzed using Mann-Whitney or Kruskal-Wallis tests. Categorial data were analyzed using the Fisher exact test. Sample sizes (*n*, number of embryos) are provided within Figure legends, along with the statistical test used in each case.

## Results

### Zebrafish Genetic Systems for Cardiovascular Manipulations: Vascular Endothelial Cell Depletion, *sih*, and *vlt*

To test roles for vascular endothelial cells in the regulation or support of retinal neurogenesis and differentiation, we used *Tg(cdh5:Gal4ff)* zebrafish, in which the vascular endothelial cell-specific *cadherin5* promoter (Larson et al., 2004) drives expression of Gal4 (Bussmann et al., 2011), crossed with *Tg(UAS-E1b:Eco.NfsB-mCherry)* zebrafish, in which the Gal4-inducible UAS promoter drives expression of a fusion protein consisting of *E. coli* nitroreductase (nfsB) and mCherry (Curado et al., 2008). Doubly transgenic embryos therefore expressed the fusion protein selectively in vascular endothelial cells (Figure 1A). When treated with the prodrug metronidazole (Met), nitroreductase catalyzes the production of a cytotoxic metabolite, leading to death of the targeted cell type (Curado et al., 2008). Met-treated clutchmates carrying one transgene (or that were WT) served as controls (“Met controls|”) for any off-target effects of Met, and DMSO-treated doubly transgenic clutchmates served as additional controls (“DMSO controls”) for effects of the vehicle. DMSO has been demonstrated to have no effect on development of the hyaloid vasculature at concentrations up to 2% (Ohnesorge et al., 2019).

Vascular endothelial cells first appear adjacent to the zebrafish retina as the hyaloid vasculature begins to form around 18 hpf (Hartsock et al., 2014; Hashiura et al., 2017). *Tg(cdh5:GAL4ff;UAS-E1b:EcoNfsB-mCherry)* embryos were treated with Met at 12 hpf. Our initial goal was to disrupt the formation of any ocular vasculature. DMSO controls at 48 hpf showed mCherry+ ring vessels around the lens along with nrv, vrv and drv, as well as a network of hyaloid vessels under the lens (Figure 1A). In contrast, Met-treated doubly transgenic embryos had extremely reduced/absent mCherry expression surrounding the lens at 48 hpf (Figure 1B). Occasional remnants of the nrv and a few patches of mCherry around the lens were present in a few of these embryos, indicating that most of the ocular vasculature was depleted at 48 hpf. Vessels remained disrupted through 72 hpf, although sporadic expression of mCherry was seen within the eye at this age (Figures 1C,D). We observed that the mCherry reporter occasionally showed mosaic expression, and so to confirm the presence of vessels and blood in controls, and their absence or near-absence in doubly transgenic Met-treated embryos, we also imaged using the *kdrl:GFP* and *gata1:dsRed* transgenic backgrounds. These studies confirmed the severe disruption of the ocular vasculature, and lack of circulating blood cells (Supplementary Figure 1).

We further verified the presence vs. absence of circulation in Met-treated doubly transgenic embryos at 36, 42, 45, 48, and 72 hpf by visualizing circulating materials in eyes of live embryos using brightfield and Nomarski optics. In control embryos, and in some Met-treated doubly transgenic embryos examined at 36, 42, and 45 hpf, circulating materials were observed entering the ring vessels around the lens through the nrv and leaving the retina through vrv and drv (Supplementary Movies 1, 2 and Table 1). In contrast, no evidence of circulation or circulating materials was seen in Met-treated doubly transgenic embryos examined at 48 or 72 hpf, despite the presence of a heart beat, suggesting that intact vasculature was missing from the eye over that time frame (Supplementary Movie 3 and Table 1). We therefore focused our analyses of retinal development over a timeframe beginning at 48 hpf and ending at 72 hpf. For the remainder of this paper, Met-treated doubly transgenic embryos are referred to as vascular endothelial cell-depleted.

**TABLE 1:** Time-course of experimental depletion of vascular endothelial cells in the zebrafish eye.

**Experimental condition (n)**	**% embryos with circulation**				
	**36 hpf**	**42 hpf**	**45 hpf**	**48 hpf**	**72 hpf**
*cdh5:gal4; UAS:nfsB-mCherry*+DMSO (DMSO Control) (8)	100	100	100	100	100
Single transgenic or WT+Met (Met Control) (8)	100	100	100	100	100
*cdh5:gal4; UAS:nfsB-mCherry*+Met (Endothelial Cell-Depleted) (10)	100	60	20	0	0

To test roles for circulating factors in the regulation or support of retinal neurogenesis and differentiation, we used *sih* mutants lacking heart contractility, and therefore with no blood flow (Clark et al., 2011). When visualized with the *kdrl:eGFP* transgenic background, the ocular vessels were clearly present but appeared collapsed and the superficial system was not fully formed (Kaufman et al., 2015; Figures 1E,F). We also utilized *vlt* mutants lacking blood cells of the erythroid lineage, including erythrocytes (Lyons et al., 2002). When visualized with the *kdrl:eGFP* background, the ocular vasculature of *vlt* mutants appeared normal in comparison with normal siblings (Figures 1G,H).

### Lens and Eye Sizes Are Reduced in Endothelial Cell-Depleted and *sih*−/− Embryos

As an initial assessment of overall effects of endothelial cell depletion, we measured Feret’s diameter of the lenses and the eyes of embryos at 48 and/or 72 hpf. At 48 hpf, we did not find any difference in lens or eye diameter, between control and vascular endothelial cell-depleted embryos (Figures 2A,C). However, when sampled at 72 hpf, both lens and eye diameter were smaller in vascular endothelial cell-depleted embryos than in control embryos (Figures 2B,C; *p* < 0.001 for eye and *p* < 0.01 for lens), as the eyes of these experimental embryos apparently did not grow (remaining ∼275 μm), while those of their control counterparts did (Figures 2A-C). Note that the embryos depleted of vascular endothelial cells also displayed swollen epicardial sacs (Figures 2A,B). Disruption of the cardiovascular system from 48-72 hpf therefore may be sufficient to result in these ocular and epicardial defects.

To gain insight as to whether reduced eye size was due to a deficiency in factors provided by vascular endothelial cells, vs. factors derived from the circulation, we measured Feret’s diameter of lenses and eyes of *sih* mutants (lacking circulation, but with endothelial cells present), in comparison with their normal siblings. At 48 hpf, both eyes and lenses of *sih* mutants were smaller than their normal siblings (Figure 2D; *p* < 0.001 for both). These differences persisted at 72 hpf (Figure 2E; *p* < 0.001 for both), although the eyes and lenses of *sih* mutants appeared to enlarge slightly over this time. These findings suggest that factors from the circulation may indeed influence the embryonic growth of these tissues. To determine whether cells of the erythroid lineage specifically are in any way required for eye growth, we measured eyes of *vlt* mutants, which lack these cells, vs. their wild-type siblings, at 72 hpf. The appearance of the *vlt−/−* eyes was superficially normal, and there were no differences in diameter in *vlt−/−* vs. wild-type eyes (Figure 2F; *p* = 0.113), suggesting that the factor(s) needed from the circulation do not depend on the presence of cells of the erythroid lineage. Of particular importance, erythrocytes and their functions (gas transport), appear nonessential for eye growth up to 72 hpf in the zebrafish.

### Abnormal Histology of Retinas in Endothelial Cell-Depleted and *sih*−/− Embryos

**FIGURE 3:**
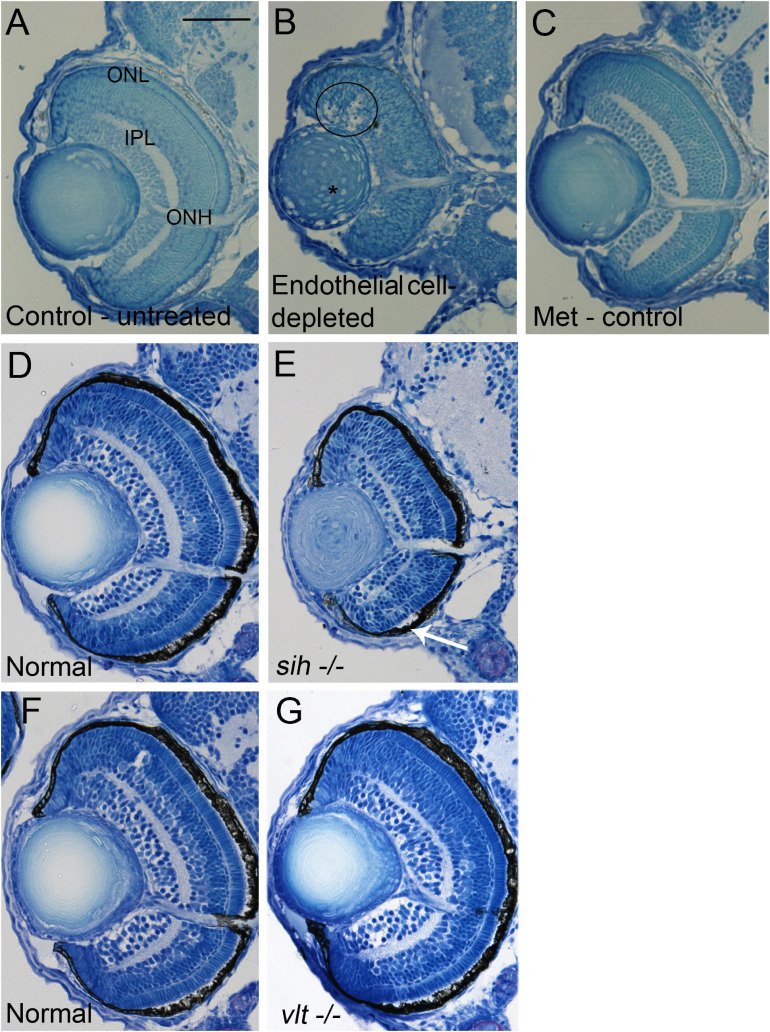
Retinal histology in cardiovascular disruption model systems. **(A,C)** Plastic, methylene blue/Azure II-stained sections of doubly-transgenic (*cdh5:gal4; UAS:nfsB-mCherry*), DMSO-treated (DMSO Control, *n* = 6; **(A)**; Met-treated (Endothelial Cell-Depleted, *n* = 8; **(B)**; and Met-treated clutchmates (Met Control, *n* = 2; **(C)** at 72 hpf. Control retinas show defined nuclear and plexiform layers and photoreceptor apical processes, while endothelial cell-depleted retinas are disorganized, with poorly defined layers, pale, acellular areas containing pyknotic, darkly-stained nuclei (example appears within black circular profile in **(B)**. Interior of lens contains clusters of nuclei (*). **(D,E)** Plastic sections of normal clutchmates (*n* = 5; **(D)** and *sih–/–* embryos (*n* = 13; **E**) at 72 hpf. The inner plexiform layer appears reduced in thickness in *sih–/–* compared to normal, and there is evidence of photoreceptor apical processes only within a ventral patch (white arrow in **(E)**. **(F,G)** Plastic sections of normal clutchmates (*n* = 5; **(F)** and *vlt–/–* embryos (*n* = 9; **(G)** Histology of *vlt–/–* retina appears similar to normal siblings at 72 hpf. ONL, outer nuclear layer; IPL, inner plexiform layer; ONH, optic nerve head. Scale bar (in **(A)**, applies to all) = 50 μm. Embryos in **(A–C)** were PTU-treated; those in D-G were not.

We next examined retinal histology in the vascular endothelial cell-depleted condition, in methylene blue stained plastic sections (Figures 3A-C) and in hematoxylin and eosin (H&E) stained cryosections (Supplementary Figure 2A-C) derived from 72 hpf embryos. Controls derived from *cdh5:gal4 X UAS:NfsB-mCherry* crosses, all displayed three (hematoxylin+) retinal nuclear layers separated by clearly defined (eosin+) plexiform layers, a prominent optic nerve head (ONH), and spindle-shaped retinal cells at the periphery, where the ciliary marginal zone (CMZ) of retinal stem and progenitor cells resides (Raymond et al., 2006; Figures 3A,C and Supplementary Figures 2A,C). The endothelial cell-depleted retinas showed reduced organization of the retinal layers, such that it was challenging to delineate any layers in many of the samples (Figure 3B and Supplementary Figure 2B). An optic nerve head was present, but reduced in thickness. Pyknotic, darkly-stained nuclei were present within the retina, generally surrounded by very weakly stained gaps in retinal tissue, providing evidence of cell death. These features appeared most prominently but were not limited to the inner region of dorsal retina. The CMZ was more difficult to discern as distinct from the remainder of the retina. Lenses contained nuclei within the central lens. We observed expanded regions of eosin+ material in the space between the retina and lens, and in the region peripheral to the CMZ (Supplementary Figure 2B). It is possible that the eosin+ material represents cytoplasmic debris associated with the death of endothelial cells of the hyaloid and radial vessels.

To test whether the disorganized retinal phenotype is related to a requirement for vascular endothelial cells vs. factors from the circulation, we examined histological preparations of *sih* and *vlt* mutants, and their normal siblings, collected at 72 hpf. The *sih* mutant retinas displayed lamination at this age, although with a thinner IPL, and little evidence of photoreceptor apical processes in comparison with normal siblings. Nuclei of horizontal cells, and the outer plexiform layer also appeared greatly reduced. Additionally, lenses of *sih* mutants were not normally differentiated at this stage (Figures 3D,E and Supplementary Figures 2D,E). In contrast, the *vlt* mutant retinas appeared strikingly similar to those of their normal siblings, with normal lamination and appearances of each retinal layer (Figures 3F,G and Supplementary Figure 2F,G). Collectively these findings indicate that the disorganized retinal phenotype of *cloche* (Dhakal et al., 2015) and that of vascular endothelial cell-depleted embryos, are more likely related to the absence of endothelial cells rather than the lack of circulating materials. However, the lack of circulating materials other than erythrocytes may have deleterious consequences for the growth/maturation of photoreceptors and for the neurogenesis/differentiation of retinal neurons that results in the growth of plexiform layers.

### Increased Cell Death in Retinas of Endothelial Cell-Depleted Embryos

**FIGURE 4:**
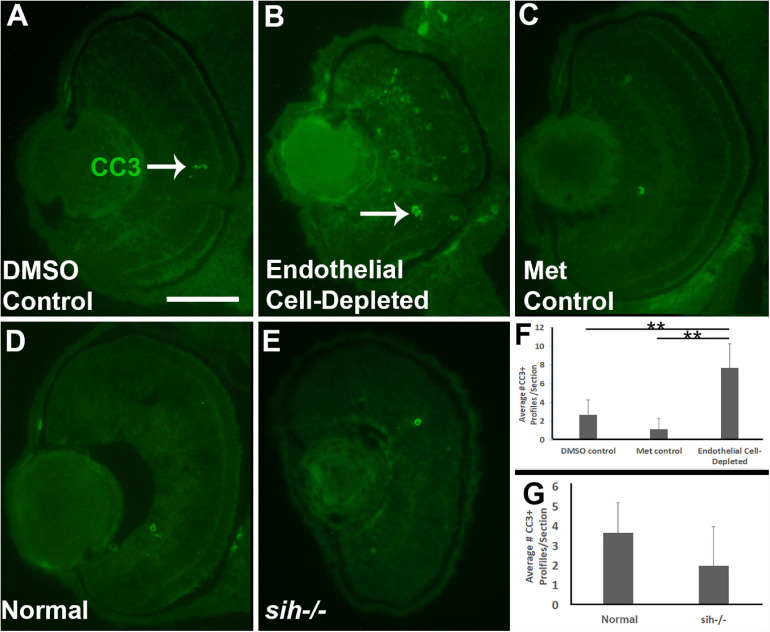
Retinal cell death in cardiovascular disruption model systems. **(A,C)** Cryosections of doubly-transgenic (*cdh5:gal4; UAS:nfsB-mCherry*), DMSO-treated (DMSO Control; **(A)**; Met-treated (Endothelial Cell-Depleted; **(B)**; and Met-treated clutchmates (Met Control; **(C)** at 72 hpf, stained with anti-activated caspase 3 (α-CC3). Control retinas show very few CC3+ profiles (arrow in A), while endothelial cell-depleted retinas display more widespread CC3+ stained-material (arrow in B), indicating cell death. **(D,E)** Cryosections of normal clutchmates **(D)** and *sih–/–* embryos **(E)**, stained with α-CC3. Cell death appears not to be widespread in retinas of *sih–/–*. Scale bar (in **A**, applies to **A–E**) = 50 μm. **(F,G)** Quantification of CC3+ profiles in retinas of endothelial cell-depleted vs. control embryos (**F**; **p < 0.01, ANOVA with Tukey *post hoc*; *n* = 6 per condition) and *sih–/–* vs. WT embryos (**G**; *p* = 0.16, Student’s *t*-test; *n* = 6 per condition).

Reduced embryonic eye size and histological disruptions may be related to increased cell death, reduced proliferation, and/or reduced cell differentiation within tissues of the eye (Kashyap et al., 2007). Therefore, we tested for cell death in vascular endothelial cell-depleted embryos, using anti-cleaved caspase-3 (αCC3) to label cells undergoing CC3-mediated apoptosis. We detected a greater number of CC3+ cells in vascular endothelial cell-depleted embryos in comparison with controls sampled at 72 hpf (*p* < 0.01) (Figures 4A–C,F). CC3+ profiles were present in all retinal layers, indicating that the dying cells were not simply the targeted endothelial cells. Therefore, increased cellular death in the retinas of zebrafish embryos lacking vascular endothelial cells is potentially one of the reasons underlying the observed reduced eye size.

We next counted CC3+ profiles in the retinal cryosections obtained from *sih* mutants vs. their normal siblings (Figures 4D,E). Interestingly, numbers of apoptotic retinal cells were not different in the *sih* mutants in comparison with normal siblings at 72 hpf (*p* = 0.148; Figure 4G), suggesting that increased cell death may not explain reduced eye size in these embryos. The *vlt* mutants were not examined for evidence of cell death, because they did not show reduced eye size.

### Reduced Cell Proliferation in Retinas of Endothelial Cell-Depleted and *sih*−/− Embryos

**FIGURE 5:**
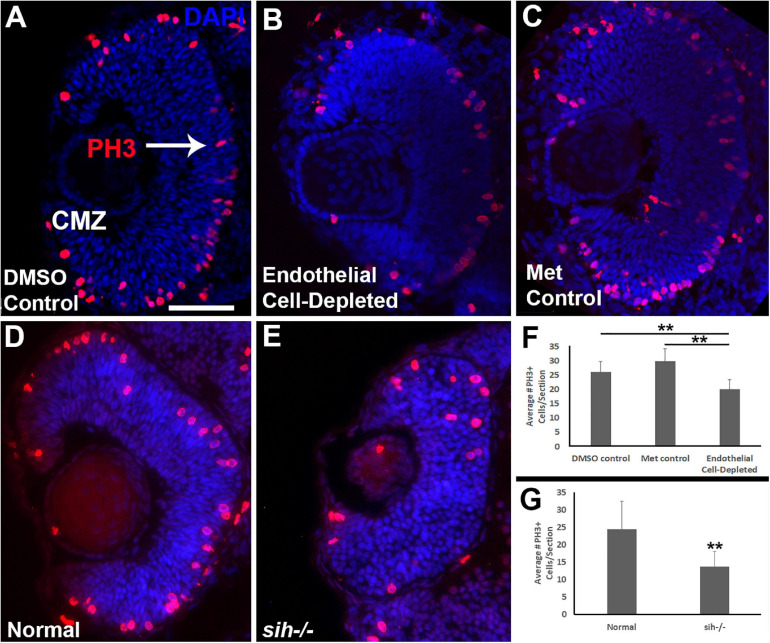
Retinal cell proliferation in cardiovascular disruption model systems. **(A,C)**. Cryosections of doubly-transgenic (*cdh5:gal4; UAS:nfsB-mCherry*), DMSO-treated (DMSO Control; **(A)**; Met-treated (Endothelial Cell-Depleted; **(B)**; and Met-treated clutchmates (Met Control; **(C)** at 48 hpf, stained with anti-phosphohistone H3 (PH3; red fluorescence) and counterstained with DAPI (blue). Control retinas show numerous PH3+ profiles, particularly within apical retina (arrow in **A**) and the ciliary marginal zone (CMZ). Endothelial cell-depleted retinas also display apically-positioned PH3+ cells that appear to be reduced in number **(B)**. **(D,E)** Cryosections of normal clutchmates **(D)** and *sih–/–* embryos **(E)**, stained with α-PH3 and DAPI. Normal retinas show numerous PH3+ profiles while *sih–/–* retinas display fewer PH3+ cells. Scale bar (in **A**, applies to **A–E**) = 50 μm. **(F,G)** Quantification of PH3+ profiles in retinas of endothelial cell-depleted vs. control embryos (**F**; **p < 0.01 ANOVA with Tukey *post hoc*; *n* = 9 DMSO, 11 Met control, and 14 Endothelial cell-depleted) and *sih–/–* vs. normal siblings (**G**; **p < 0.01, Student’s *t*-test; *n* = 10 normal, 11 *sih–/–*).

Microphthalmia can also be the consequence of reduced retinal cell proliferation; therefore, we used anti-phosphohistone-H3 (PH3) antibody to label cells in M-phase in 48 hpf embryos. In control embryos, mitotic cells (PH3+) were present predominantly in the apical retina (future ONL), with additional PH3+ cells within the CMZ (Figures 5A,C). In vascular endothelial cell-depleted embryos, PH3+ cells were also detected in these locations (Figure 5B), but in reduced numbers (Figure 5F; *p* < 0.01) indicating that reduced proliferation likely also contributes to the lack of eye growth between 48 and 72 hpf.

Because the *sih* mutant eyes were also reduced in size, and this did not appear to be related to increased cell death, we evaluated cell proliferation in these embryos as well. Numbers of PH3+ retinal cells were reduced in *sih* mutants in comparison with their normal siblings (Figures 5D,E,G; *p* < 0.01), suggesting that factors from the circulation influence retinal cell proliferation within embryos, and this reduced proliferation may in part underlie reduced eye size. The *vlt* mutants were not examined for cell proliferation, because they did not show reduced eye size.

### Reduced Photoreceptor Differentiation in Retinas of Endothelial Cell Depleted and *sih*−/− Embryos

**FIGURE 6:**
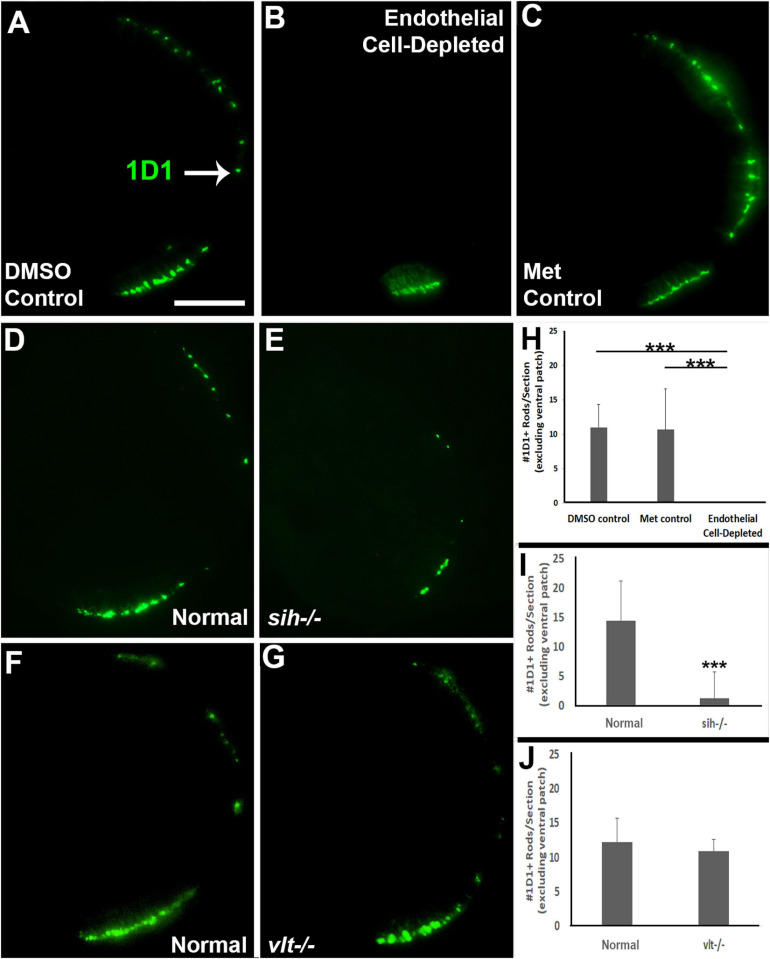
Rod photoreceptors in cardiovascular disruption model systems. **(A,C)** Cryosections of doubly-transgenic (*cdh5:gal4; UAS:nfsB-mCherry*), DMSO-treated (DMSO Control; **(A)**; Met-treated (Endothelial Cell-Depleted; **(B)**; and Met-treated clutchmates (Met Control; **C**) at 72 hpf, stained with 1D1, which labels Rhodopsin. Control retinas show numerous rods **(A**, arrow), while endothelial cell-depleted retinas display only a patch of rods in ventral retina **(B)**. **(D,E)** Cryosections of normal clutchmates **(D)** and *sih–/–* embryos **(E)** at 72 hpf, stained with 1D1. Normal retinas show numerous 1D1+ profiles while *sih–/–* retinas show a reduced number of rods. **(F,G)** Cryosections of normal clutchmates **(F)** and *vlt–/–* embryos **(G)** at 72 hpf, stained with 1D1, show similar patterns of staining. Scale bar (in **A**, applies to **A–G**) = 50 μm. **(H,J)** Quantification of 1D1+ rods outside the ventral patch, in retinas of endothelial cell-depleted vs. control embryos (**H**; ***p < 0.001, Kruskal-Wallis with Conover *post hoc*; *n* = 6 per condition), *sih–/–* vs. normal siblings (**I**; ***p < 0.001, Mann-Whitney test; *n* = 12 normal, 19 *sih–/–*), and *vlt–/–* vs. normal siblings (**J**; *p* = 0.55 Student’s *t*-test; *n* = 9 normal, 8 *vlt–/–*).

Reduced eye size in the vascular endothelial cell-depleted embryos and in *sih* mutants, and the disorganized retina of the former, may also be related to reduced retinal cell differentiation. Cell differentiation enlarges the eye as cells extend processes, build synaptic layers, and secrete extracellular matrix material (Kashyap et al., 2007). The period of embryonic rod and cone photoreceptor terminal mitosis and initial differentiation takes place from 48–72 hpf in zebrafish embryos (Hu and Easter, 1999; Suzuki et al., 2013), and histological preparations of both vascular endothelial cell-depleted embryos and *sih* mutants displayed thinned or missing photoreceptor apical processes (Figure 3 and Supplementary Figure 2). We used 1D1 antibody to label Rhodopsin in rod photoreceptors, and zpr-1 antibody to examine long and middle wavelength-sensitive double-cone photoreceptors, at 72 hpf. Rods were most abundant within a ventral patch of the ONL, but also more sparsely present throughout the ONL in control embryos (Figures 6A,C). In contrast, rods were limited to ventral retina as a single patch of cells in vascular endothelial cell-depleted embryos (Figure 6B). Numbers of 1D1+ rods outside the ventral patch were greatly reduced in comparison with controls (Figure 6H; *p* < 0.001). Interestingly, rods were also highly reduced in number in *sih* mutants in comparison with their normal siblings (Figures 6D,E,I; *p* < 0.001). These findings suggest that at least some of the factors required for normal rod differentiation are missing in both situations, and therefore may be derived from the circulation rather than exclusively from vascular endothelial cells. As an additional test for the contribution of erythroid lineage blood cells, we also stained cryosections of *vlt−/−* vs. normal clutchmates with 1D1. Numbers of rods in the *vlt* mutants were similar to those of their normal siblings (Figures 6F,G,J; *p* = 0.55), indicating that the required factor(s) are not derived from the presence of erythrocytes.

**FIGURE 7:**
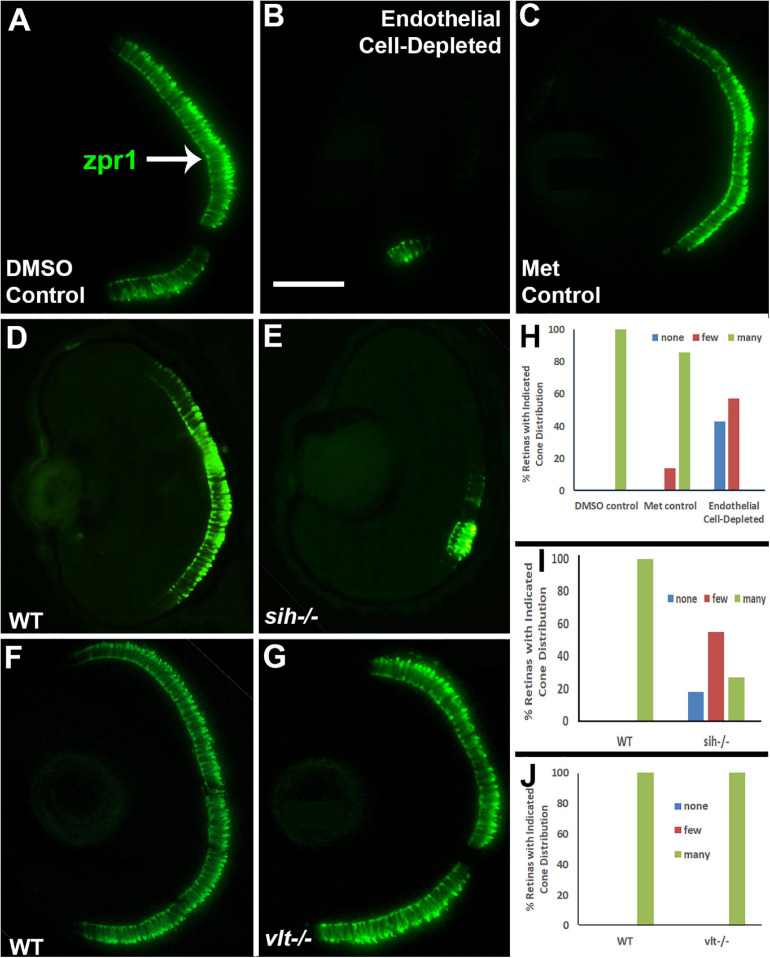
Cone photoreceptors in cardiovascular disruption model systems. **(A,C)**. Cryosections of doubly-transgenic (*cdh5:gal4; UAS:nfsB-mCherry*), DMSO-treated (DMSO Control; **(A)**; Met-treated (Endothelial Cell-Depleted; **(B)**; and Met-treated clutchmates (Met Control; **(C)** at 72 hpf, stained with zpr1, which labels red- and green-sensitive (LWS and RH2) double cones. Control retinas show numerous cones **(A**, arrow), while endothelial cell-depleted retinas display only a patch of cones in ventral retina **(B)**. **(D,E)** Cryosections of normal clutchmates **(D)** and *sih–/–* embryos **(E)** at 72 hpf, stained with zpr1. Normal retinas show numerous zpr+ profiles while *sih–/–* retinas show a reduced number of cones. **(F,G)** Cryosections of normal clutchmates **(F)** and *vlt–/–* embryos **(G)** at 72 hpf, stained with zpr1, show similar patterns of staining. Scale bar (in **B**, applies to **A–G**) = 50 μm. **(H,J)** Assessment of distribution of zpr1+ cones, in retinas of endothelial cell-depleted vs. control embryos **(H**; *p* < 0.001, Fisher exact test; *n* = 7 for each condition), *sih–/–* vs. normal siblings (**I**; *p* < 0.001, Fisher exact test; *n* = 8 normal, 11 *sih–/–*), and *vlt–/–* vs. normal siblings (**J**; *p* = 1.0, Fisher exact test; *n* = 9 normal, 11 *vlt–/–*).

Next, we analyzed differentiation of cone photoreceptors. At 72 hpf, zpr1+ double cones were present throughout the ONL of control embryos (Figures 7A,C). Within vascular endothelial cell-depleted embryos, cones were present in variable numbers, with embryos scored as having no zpr1+ cones, and others as having “some” cones (generally limited to a ventral patch; Figure 7B). Both of the control groups were predominantly scored as having “many” zpr1+ cones, while endothelial cell-depleted embryos were scored as either having “none” or “few” (Figure 7H; *p* < 0.001, Fisher exact test). In *sih* mutants, cones were also present in variable numbers, but in general, reduced in comparison with their normal siblings (Figures 7D,E,I; *p* < 0.001), while *vlt* mutants displayed a normal distribution of zpr1+ cones (Figures 7F,G,J; *p* = 1.0). In summary, differentiation of rod and cone photoreceptors was greatly reduced in the absence of vascular endothelial cells, also highly reduced in the absence of circulating materials, but proceeded normally in the absence of circulating erythrocytes.

### Disorganized Plexiform Layers and RGCs in Retinas of Endothelial Cell-Depleted Embryos and Thinned Plexiform Layers in *sih*−/− Retinas

**FIGURE 8:**
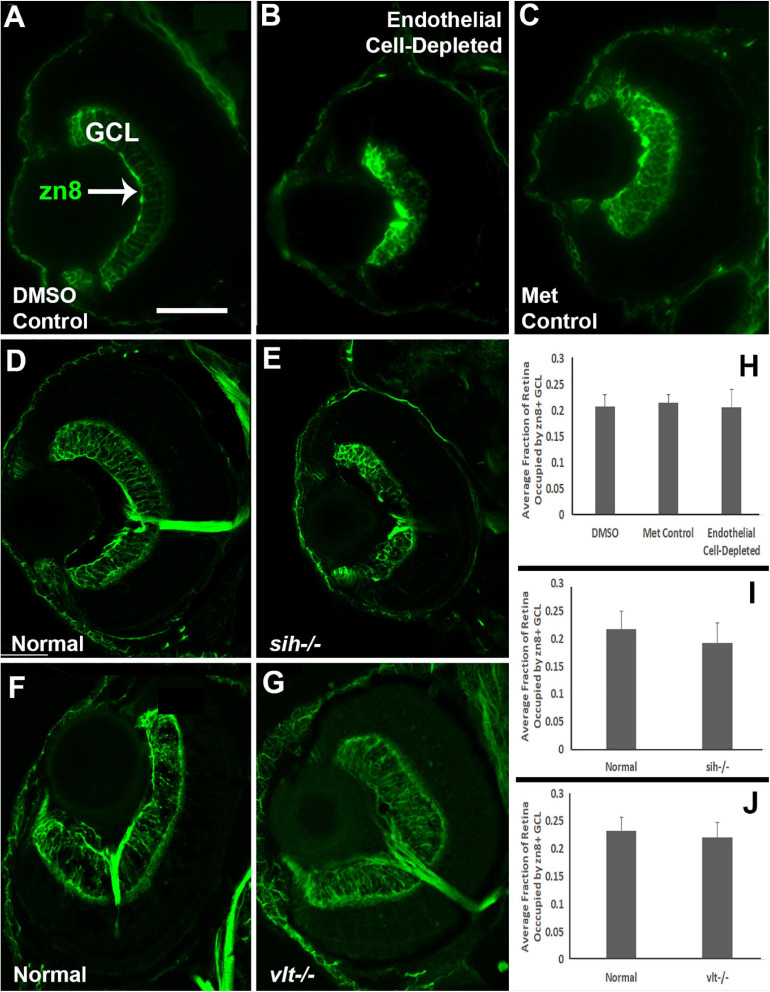
Retinal ganglion cells in cardiovascular disruption model systems. **(A,C)** Cryosections of doubly-transgenic (*cdh5:gal4; UAS:nfsB-mCherry*), DMSO-treated (DMSO Control; **(A)**; Met-treated (Endothelial Cell-Depleted; **(B)**; and Met-treated clutchmates (Met Control; **(C)** at 72 hpf, stained with zn8, which labels DM-Grasp/Neurolin/Alcama, present on neurons growing long axons, and more weakly expressed in neuroepithelial cells. Control retinas show a well-defined layer of retinal ganglion cells within the ganglion cell layer (GCL) **(A**, arrow), while endothelial cell-depleted retinas display a reduced and disorganized GCL **(B)**. **(D,E)** Cryosections of normal clutchmates **(D)** and *sih-/-* embryos **(E)** at 72 hpf, stained with zn8. Normal retinas show a clearly stained zn8+ GCL **(D)** and *sih–/–* retinas also show clear staining, with the GCL apparently reduced in thickness and slightly disorganized **(E)**. **(F,G)** Cryosections of normal clutchmates **(F)** and *vlt–/–* embryos **(G)** at 72 hpf, stained with zn8; the GCL of *vlt–/–* retina appears normal. **(H,J)** Quantification of fraction of retina occupied by the GCL (GCL/retina ratio), in retinas of endothelial cell-depleted vs. control embryos (**H**; *p* < 0.814, Kruskal-Wallis test, *post hoc* analysis not justified; *n* = 7 DMSO controls, 8 Met controls, 10 endothelial cell-depleted), *sih–/–* vs. normal siblings (**I**; *p* = 0.147, Mann-Whitney test; *n* = 12 normal, 11 *sih–/–*), and *vlt–/–* vs. normal siblings (**J**; *p* = 0.459, Mann-Whitney test; *n* = 13 normal, 21 *vlt–/–*). Scale bar (in A, applies to all) = 50 μm.

Cells of the retinal ganglion cell layer (GCL) are the first retinal neurons to exit the cell cycle in zebrafish embryos (Hu and Easter, 1999). We evaluated differentiation specifically of ganglion cells at 72 hpf by labeling with the zn8 antibody, which stains Neurolin/DM-Grasp, present on neurons that are growing long axons (Tallafuss and Eisen, 2008). A differentiating, zn8+ GCL was evident in controls (Figures 8A,C), but was irregular in appearance and reduced in size in vascular endothelial cell-depleted embryos (Figure 8B). The *sih* mutants displayed more robust zn8 staining of the GCL than the vascular endothelial cell-depleted embryos, with perhaps some minor abnormalities in the form of irregular staining (Figures 8D,E), and the *vlt* mutants showed apparently normal zn8 staining of RGCs (Figures 8F,G). We next measured the perimeters of the zn8+ GCL, and of the retina, and then calculated the GCL/retina ratios. These ratios were not different for each comparison (Figures 8H,J; *p* = 0.814, 0.147, 0.459, respectively). Therefore, the GCLs within endothelial cell-depleted embryos and *sih* mutants were not disproportionately affected by the vascular and circulation disruption with respect to the remainder of the retina.

**FIGURE 9:**
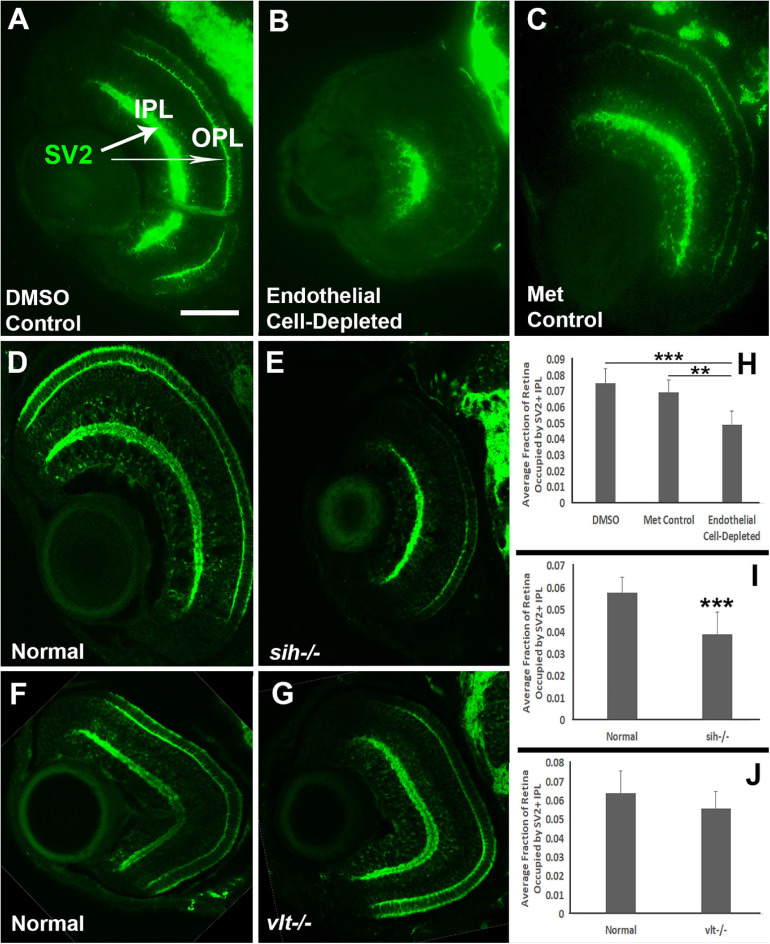
Plexiform layers in cardiovascular disruption model systems. **(A,C)** Cryosections of doubly-transgenic (*cdh5:gal4; UAS:nfsB-mCherry*), DMSO-treated (DMSO Control; **(A)**; Met-treated (Endothelial Cell-Depleted; **(B)**; and Met-treated clutchmates (Met Control; **(C)** at 72 hpf, stained with anti-Synaptic vesicle 2 (SV2), which stains synaptic terminals in inner and outer plexiform layers (IPL, OPL). Control retinas show well-defined plexiform layers (**A**, arrows), while endothelial cell-depleted retinas display a reduced and disorganized IPL and no clear OPL **(B)**. **(D,E)** Cryosections of normal clutchmates **(D)** and *sih–/–* embryos **(E)** at 72 hpf, stained with anti-SV2. Normal retinas show well-defined plexiform layers **(D)**, and *sih–/–* retinas also show well-defined plexiform layers, apparently reduced in thickness, with OPL also limited to the ventral half of the retina. Some of the reduced size appears proportional to the overall reduced eye size **(E)**. **(F,G)** Cryosections of normal clutchmates **(F)** and *vlt–/–* embryos **(G)** at 72 hpf, stained with anti-SV2; the plexiform layers of *vlt–/–* retina appear normal. **(H,J)** Quantification of fraction of retina occupied by the IPL (IPL/retina ratio), in retinas of endothelial cell-depleted vs. control embryos (**H**; ***p < 0.001, **p < 0.01, Kruskal-Wallis with Conover *post hoc*; *n* = 8 DMSO control, 6 Met control, 9 endothelial cell-depleted), *sih–/–* vs. normal siblings (**I**; ***p < 0.001, Mann-Whitney test; *n* = 11 normal, 18 *sih–/–*), and *vlt–/–* vs. normal siblings (**J**; *p* = 0.0466, Mann-Whitney test; *n* = 15 for each condition). Scale bar (in A, applies to A-G) = 50 μm.

We also analyzed the presence and organization of synaptic layers in vascular endothelial cell-depleted embryos and *sih* mutants, using the presynaptic terminal marker, anti-SV2. In control embryos, SV2 stained the IPL and some associated cellular processes, and the OPL (Figures 9A,C). In vascular endothelial cell-depleted embryos, the IPL was diffusely stained by anti-SV2 in the radial orientation and appeared restricted to central retina, suggesting that synapse formation was likely affected, and that some synaptic terminals were misplaced (Figure 9B). A clearly-defined OPL was not visible in vascular endothelial cell-depleted embryos (Figure 9B). In normal siblings of *sih* mutants, SV2+ plexiform layers were distinct at 72 hpf (Figure 9D). The *sih* mutants also showed distinct, SV2+ plexiform layers, but these appeared thinned in comparison with normal clutchmates (Figure 9E), apparently in proportion to the reduced overall size of the eye. SV2 labeling also appeared limited to central retina, although not as limited as in the endothelial cell-depleted embryos. The *vlt* mutants displayed apparently normal staining of plexiform layers with anti-SV2 (Figures 9F,G). We next measured the perimeters of the SV2+ IPL, and of the retina, and then calculated IPL/retina ratios. Endothelial cell-depleted embryos showed reduced ratios in comparison with controls (Figures 9H; *p* < 0.01), and *sih* mutants also showed reduced ratios in comparison with normal siblings (Figure 9I; *p* < 0.001), while ratios of *vlt* mutants were not different from their normal siblings (Figure 9J; *p* = 0.047). Disruption of vasculature and/or circulation therefore results in reduced development of the retinal IPL.

### Impaired Müller Glia Differentiation but Normal Numbers of Microglia in Cardiovascular Disruption Models

**FIGURE 10:**
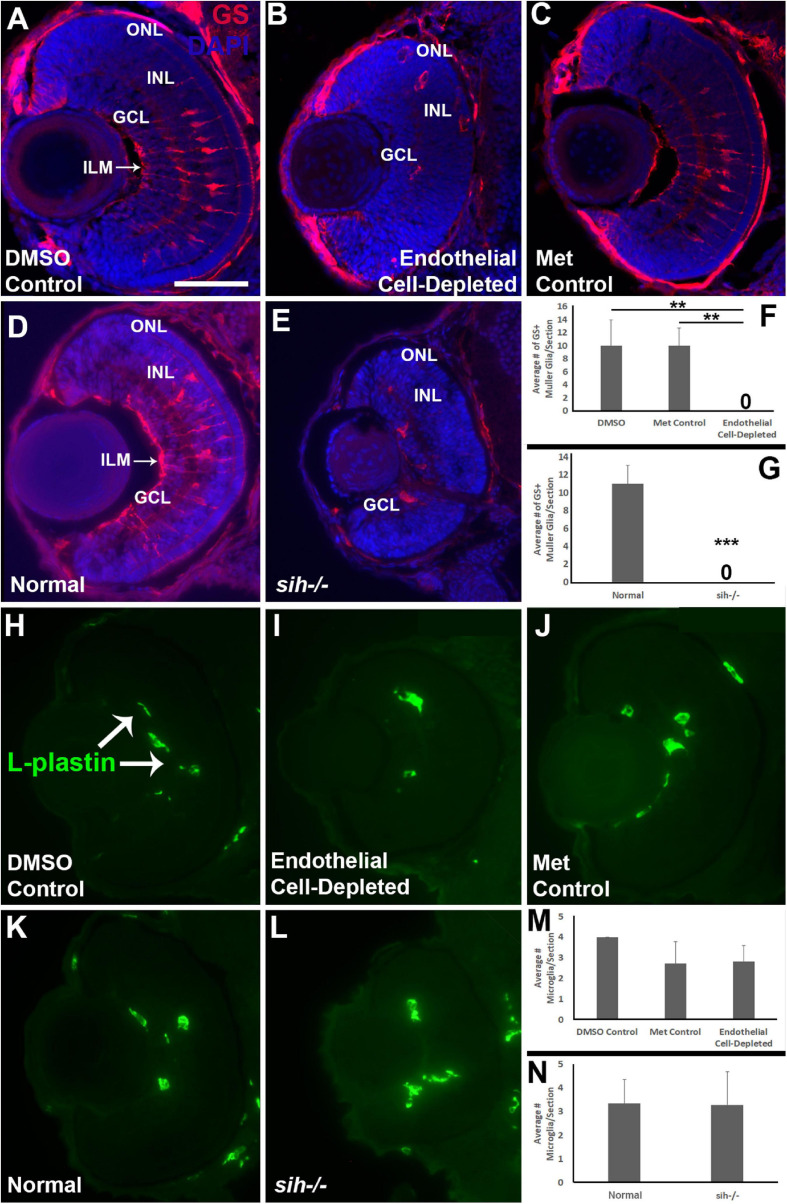
Müller glia and microglia in cardiovascular disruption model systems. **(A–C)** Cryosections of doubly-transgenic (*cdh5:gal4; UAS:nfsB-mCherry*), DMSO-treated (DMSO Control; **(A)**; Met-treated (Endothelial Cell-Depleted; **(B)**; and Met-treated clutchmates (Met Control; **(C)** at 72 hpf, stained with anti-Glutamine synthetase (GS; red fluorescence); GS is present within Müller glia, and counterstained with DAPI (blue). Control retinas show GS+ cell bodies within the INL, and GS+ Müller glial processes spanning the retina with a clear radial orientation and endfeet at the inner limiting membrane (ILM, arrow in A), while endothelial cell-depleted retinas display reduced GS staining, GS+ cell bodies in layers other than the INL, and a lack of GS+ endfeet at the ILM **(B)**. **(D,E)** Cryosections of normal clutchmates **(D)** and *sih–/–* embryos **(E)** at 72 hpf, stained with anti-GS and DAPI. Normal retinas show normally-patterned GS+ Müller glial endfeet, cell bodies, and radial processes. The *sih–/–* retinas show far fewer GS+ cell bodies, and these are not positioned within the INL. **(F,G)** Quantification of GS+ cells with Müller glial morphology shows significantly fewer following endothelial cell depletion [**(F)**; *p* < 0.01; Kruskal-Wallis with Conover *post hoc* test; *n* = 7 DMSO, 5 Met control, 6 endothelial cell-depleted], and in *sih–/–* compared with normal clutchmates [**(G)**; ***p < 0.001; Mann-Whitney test, *n* = 8 for each condition] **(H,J)** Cryosections of doubly-transgenic (*cdh5:gal4; UAS:nfsB-mCherry*), DMSO-treated (DMSO Control; **(H)**; Met-treated (Endothelial Cell-Depleted; **I**); and Met-treated clutchmates [(Met Control; **(J)**] at 72 hpf, stained with anti-L-plastin, which is a pan-leukocyte marker and stains microglia. Control retinas contain L-plastin+ microglia within the inner retina **(H**, arrows), and endothelial cell-depleted retinas also show microglia present within the inner retina (**I**). **(K,L)** Cryosections of normal clutchmates **(K)** and *sih–/–* embryos **(L)** at 72 hpf, stained with L-plastin, show similar patterns of staining. Scale bar (in A, applies to all images) = 50 μm. **(M,N)** Quantification of L-plastin+ microglia, in retinas of endothelial cell-depleted vs. control embryos [**(M)**; *p* = 0.15; ANOVA with Tukey *post hoc*; *n* = 9 for each condition)], and *sih–/–* vs. normal siblings (**N**; *p* = 0.47; Student’s *t*-test; *n* = 7 for each condition).

Müller glia are the principal glial cells of the retina, providing structural integrity and spanning the radial thickness of the retina by extending endfeet to the inner and outer limiting membranes (Newman and Reichenbach, 1996). We used anti-Glutamine synthetase (GS) antibody, and anti-glial fibrillary acidic protein (GFAP) antibody, zrf1, to label Müller glia in the zebrafish retina at 72 hpf. The control embryos showed Müller glia with GS+, GFAP+ endfeet at inner limiting membrane (ILM) at the vitreal surface, GS+ cell bodies positioned within the INL, and GS+, GFAP+ radial processes (Figures 10A,C and Supplementary Figure 3A,C). In contrast, vascular endothelial cell-depleted embryos had far fewer GS+ cell bodies (Figure 10B), and highly reduced GFAP staining of Müller glia (Fig. S3B). Radial processes and a GS+ ILM were not clearly visible (Figure 10B and Supplementary Figure 3B), and many GS+ cell bodies were not localized to the INL (Figure 10B). We were unable to locate in the endothelial cell-depleted embryos GS+ cell bodies that had normal Müller glial morphology (Figure 10F; *p* < 0.01). *sih* mutants also only rarely showed GS+ cell bodies, and these were not localized to the INL (Figures 10D,E,G, *p* < 0.001). Further, GFAP staining was difficult to detect within the *sih−/−* retinas, and when visible was primarily, although not exclusively, located at or near the optic nerve head (Supplementary Figure 3D,E). These findings suggest that Müller glial differentiation requires normal vasculature with blood flow.

Previously, we have shown that *cloche* mutant embryos have reduced numbers of microglia in their retinas at 72 hpf (Dhakal et al., 2015), leaving open the possibility that the retinal phenotype in *cloche* and potentially other vascular disruption models could be related to limited microglial function (Huang et al., 2012). In particular, microglia are required for clearance of apoptotic cells, and when they are depleted, apoptotic profiles accumulate (Blume et al., 2020). Microglia migrate from the yolk sac to the brain and retina as early macrophages around 26-30 hpf and differentiate into mature microglia at approximately 55 hpf during development in zebrafish embryos (Herbomel et al., 2001), but the pathway(s) used during the migration are not clearly known and potentially could involve the vasculature. Therefore, we tested for presence of microglia in vascular endothelial cell-depleted embryos in order to verify that their abnormal retinal phenotype was not related to absent or reduced microglia. We labeled microglia with the pan-leucocyte marker L-plastin (Blume et al., 2020; Mitchell et al., 2018) in 72 hpf embryos. L-plastin+ profiles were present predominantly within the IPL and GCL in all control embryos at 72 hpf (Figures 10H,J), consistent with reported locations of microglia in zebrafish embryos as described previously (Herbomel et al., 2001). L-plastin+ microglia were similarly detected in these locations within the retinas of vascular endothelial cell-depleted embryos (Figure 10I). We quantified the numbers of L-plastin+ microglia per section and found that there was no difference in vascular endothelial cell-depleted embryos when compared with the control embryos (Figure 10M; *p* = 0.15). Therefore, the abnormal retinal phenotype in the experimental embryos is not due to the lack of microglia. Retinas of *sih* mutants and normal clutchmates also showed microglia present in the IPL and GCL (Figures 10K,L), in similar numbers (Figure 10N; *p* = 0.47).

### Disrupted Expression of Retinal Transcription Factors in Retinas of Endothelial Cell-Depleted Embryos

**FIGURE 11:**
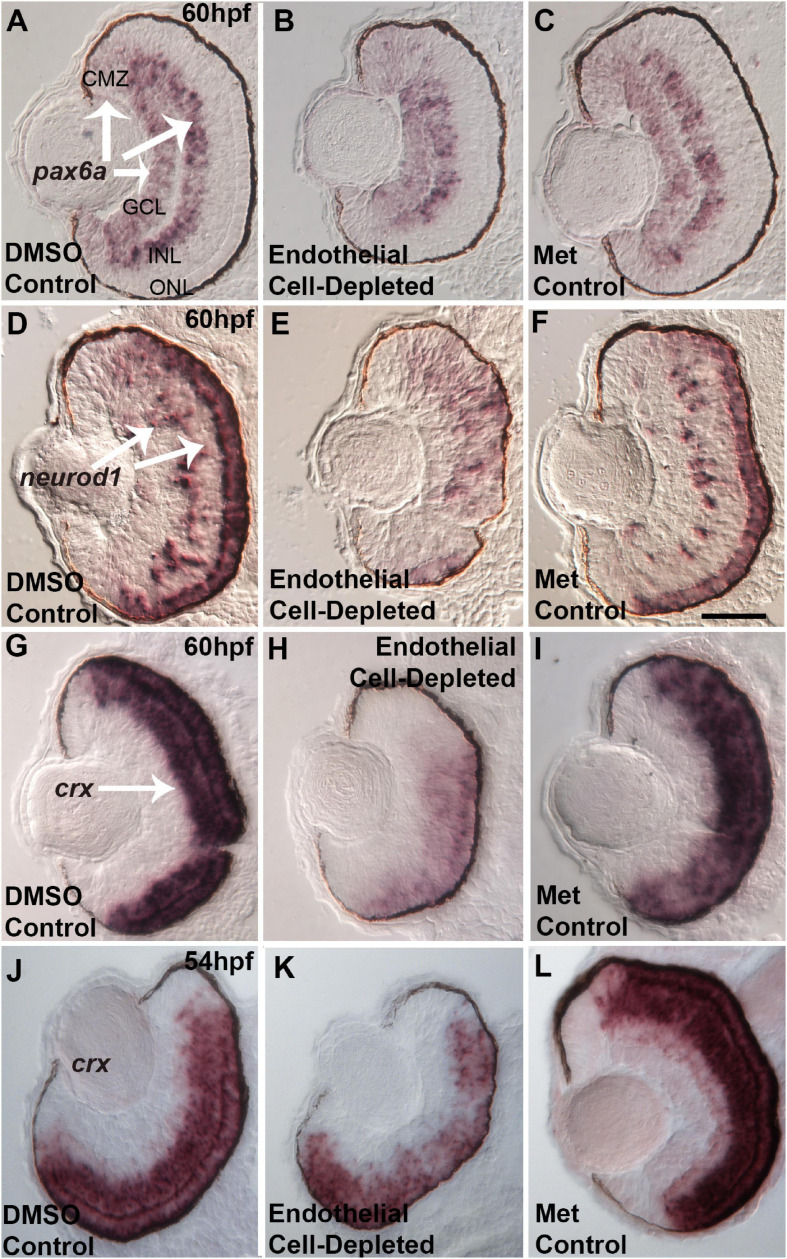
Selected retinal transcription factors in embryos depleted of vascular endothelial cells. **(A–C)** Cryosections of doubly-transgenic (*cdh5:gal4; UAS:nfsB-mCherry*), DMSO-treated (DMSO Control, n = 7; **(A)**; Met-treated (Endothelial Cell-Depleted, *n* = 9; **(B)**; and Met-treated clutchmates (Met Control, n = 8; **(C)** at 60 hpf, hybridized with probe targeting *pax6a*. Control retinas show *pax6a* expression within the ganglion cell layer (GCL), inner regions of the inner nuclear layer (INL), and ciliary marginal zone (CMZ) **(A**, arrows). Endothelial cell-depleted retinas display similar *pax6a* expression patterns **(D,F).** Same treatments as **(A,C)**, but hybridized with probe targeting *neurod1*. Control retinas show *neurod1* expression within the INL and outer nuclear layer (ONL) **(D**, arrows) **(D**, *n* = 6; E, *n* = 9; F, *n* = 9). Endothelial cell-depleted retinas display more diffuse expression, particularly in dorsal retina **(E)**. **(G–I)** Same treatments as **(A–C)**, but hybridized with probe targeting *crx*. Control retinas show *crx* expression within the outer INL and ONL **(G**, arrow). Endothelial cell-depleted retinas display weaker and more limited expression of *crx* **(H) (G)**, *n* = 9; **(H)**, *n* = 10; **(I)**, *n* = 8). **(J–L)** Same treatments and *crx* probe as G.-I., but sampled at 54 hpf. Control retinas show *crx* expression within the outer INL and ONL **(J)**. Endothelial cell-depleted retinas display patchier expression of *crx* **(K)** [**(J)**, *n* = 5; **(K)**, *n* = 6; **(L)**, *n* = 6]. Scale bar in F (applies to all) = 25 μm.

Several specific transcription factors are involved in retinal differentiation in the embryonic zebrafish. We tested the expression of selected retinal transcription factors important for retinal ganglion cells and for photoreceptors, and which show changes in expression pattern over the time of their initial differentiation, by using *in situ* hybridization and cryosectioned, 60 hpf embryos. First, we analyzed the expression of *pax6a*, which is required for overall eye development, is present in retinal progenitor cells including those of the CMZ, and eventually becomes restricted to RGCs and ACs (Macdonald et al., 1995; Nornes et al., 1998). *pax6a* was expressed strongly in the GCL and moderately in the CMZ in control embryos at 60 hpf (Figures 11A,C). Vascular endothelial cell-depleted embryos showed a similar pattern of *pax6a* expression (Figure 11B).

Next, we analyzed the expression of *neurod1*, which is required for cell cycle exit of photoreceptor progenitors in zebrafish embryos (Ochocinska and Hitchcock, 2009), and its expression becomes restricted to the ONL and scattered cells of the INL as these layers form (Nelson et al., 2008). At 60 hpf, *neurod1* was expressed in the ONL and INL in control embryos (Figures 11D,F). In contrast, *neurod1* expression was apparently reduced, diffusely distributed, and limited to smaller patches within the ONL, in vascular endothelial cell-depleted embryos (Figure 11E).

Lastly, we tested expression of *crx*. *crx* encodes a transcription factor required for expression of photoreceptor-specific genes (Shen and Raymond, 2004). *crx* mRNA transcripts were present throughout the ONL and outer INL in control embryos at 60 hpf (Figures 11G,I). However, in vascular endothelial cell-depleted embryos, expression was highly reduced, and missing in some cases from dorsal retina (Figure 11H). This *crx* expression phenotype appeared more severe than that of the avascular and bloodless *cloche* retina (Dhakal et al., 2015), which showed occasional regions of the ONL with *crx* expression reduced or missing. However, in our previous study, the *cloche* retina was sampled at 54 hpf, not 60 hpf. Therefore, we sampled vascular endothelial cell-depleted retinas at this developmental time, and observed *crx* expression patterns strikingly similar to that of *cloche* at 54 hpf (Figures 11J–L; Dhakal et al., 2015).

Reduced *neuroD1* and *crx* expression at 60 hpf in vascular endothelial cell-depleted embryos potentially contribute to the photoreceptor differentiation defects observed at 72 hpf, and/or reflect a general defect in the transition from proliferation to differentiation within the retina. These results also suggest that endothelial cells of vasculature and/or circulating factors are likely to be involved in providing signals to the developing retina, which help to maintain the expression of transcription factors required for normal retinal development.

### No Hypoxia in Endothelial Cell-Depleted Embryos

**FIGURE 12:**
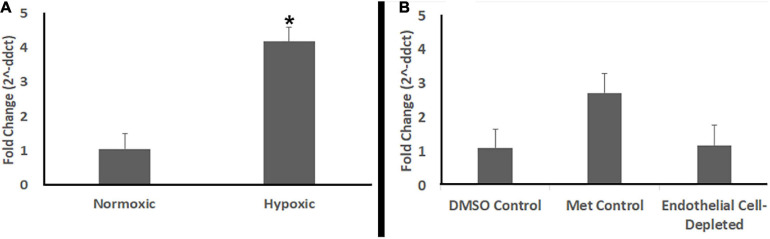
Endothelial cell-depleted embryos are not hypoxic. **(A)** Quantitative (real-time) RT-PCR (qPCR) of *prolyl-hydroxylase3* (*phd3*) transcripts shows increased *phd3* levels in experimentally hypoxic embryos vs. normoxic controls (*p < 0.05; Mann-Whitney Wilcoxon, using ddCts). **(B)** qPCR shows no increase in *phd3* levels in Met-treated, doubly-transgenic (*cdh5:gal4; UAS:nfsB-mCherry*) vs. DMSO-treated, doubly-transgenic controls (*p* = 0.89; Kruskal-Wallis with Conover *post hoc*, using ddCts; *n* = 4 per condition, where each biological replicate includes 3-4 pooled embryos).

Studies have shown that zebrafish embryos do not require active circulation of oxygen for their metabolic needs until 5 dpf or beyond (Pelster and Burggren, 1996; Jacob et al., 2002). *cloche* mutants also did not show any evidence of hypoxia at 72 hpf (Dhakal et al., 2015), although they do by 108 hpf (Takada et al., 2017). Therefore, in order to further verify that the abnormal retinal phenotype observed in vascular endothelial cell-depleted embryos was not due to hypoxia, we used quantitative real-time polymerase chain reaction (qPCR) to compare abundance of *prolyl-hydroxylase3* (*phd3*) transcripts. The expression of *phd3* is known to be upregulated in response to hypoxic conditions *in vivo* (D’Angelo et al., 2003; Dhakal et al., 2015; Takada et al., 2017). Positive control embryos exposed to hypoxic conditions showed higher levels of *phd3* transcript than normoxic controls (Figure 12A). In contrast, vascular endothelial cell-depleted embryos showed levels of *phd3* transcript that were not different from those of corresponding clutchmate control embryos (Figure 12B). Together with the lack of significant phenotype of the *vlt* mutants at 72 hpf, these findings confirm that the retinal phenotype seen in the vascular perturbation models are not due to reduced tissue oxygenation.

## Discussion

In this study we have evaluated the relative roles of vascular endothelial cells, circulating factors, and cells of the erythroid lineage, in the development of the neural retina of a vertebrate. It was possible to evaluate developmental roles for the vasculature that are independent from functions in gas transport by using zebrafish embryos, which are not dependent upon the cardiovascular system for gas transport until larval stages (Pelster and Burggren, 1996; Takada et al., 2017). Our prior study of the bloodless, avascular *cloche* mutant provided initial evidence for such developmental roles (Dhakal et al., 2015). Here, in this work, we illuminate the timeframe for these developmental roles, and uncouple functions of circulating factors, which could include blood cells and endocrine factors, from potential paracrine or cell contact-mediated signals from vascular endothelial cells.

The main findings of the present study were: (1) the zebrafish vasculature is likely required from 48 hpf to 72 hpf to support cell proliferation, cell survival, cell differentiation, and tissue organization of the developing retina; (2) retinal disorganization and cell death are likely due to a requirement for factors derived from endothelial cells; (3) some of the retinal defects in proliferation and differentiation are at least in part due to a requirement for circulating factors; (4) the disorganized retinal phenotype that results from the lack of vasculature is not due to a lack of microglia; and (5) none of the defects in retinal development that result from the lack of vasculature are due to a lack of cells of the erythroid lineage or related to hypoxia.

### Vascular Endothelial Cells Are Needed to Support Retinal Organization and Retinal Cell Survival, From 48 hpf to 72 hpf

Our approach for selective depletion of vascular endothelial cells resulted in their ablation from 48 hpf to 72 hpf, with some embryos displaying vessel depletion by 42 hpf. The strategy of treating with metronidazole at 12 hpf was intended to deplete these cells earlier in development; it is possible that the bacterial nitroreductase in the transgenic embryos was not abundantly expressed and/or fully functional until later. This outcome fortuitously provided the opportunity to study the consequences of vessel depletion over a restricted timeframe of eye development. This time corresponds with rapid angiogenesis within the hyaloid vessel network, characterized by sprouting and branching, and an increase in the complexity and density of this network (Hartsock et al., 2014). The superficial system (“radial vessels”) is almost fully established around 48 hpf, as is the primordial hindbrain channel behind the eye (Kaufman et al., 2015). Toward the end of this timeframe, the hyaloid network becomes connected to the annular “ring” vessel of the superficial network (Hartsock et al., 2014). Within the retina, RGCs and cells of the INL have exited the cell cycle and are differentiating, growing processes and establishing the plexiform layers (Hu and Easter, 1999), while photoreceptor terminal mitoses take place and photoreceptors differentiate in a wave that begins in ventral retina and proceeds nasally, then dorsally, and then temporally (Raymond et al., 1995; Hu and Easter, 1999; Suzuki et al., 2013). Müller glia also become established over this developmental period, as their nuclei become properly positioned within the INL, they begin to express GFAP, and they form endfeet associations with vessels of the hyaloid (MacDonald et al., 2015; Charlton-Perkins et al., 2019). The present study shows that depletion of vessels from ∼48 hpf to 72 hpf clearly interferes with these developmental processes within the retina. The plexiform layers do not become established, and differentiation of photoreceptors and Müller glia are both severely impaired. There is an accumulation of apoptotic bodies within the retina, and this is not due to a lack of microglia that can clear them. Collectively these defects result in the failure of the eye to grow over the time of vessel depletion. The striking similarity of the vessel depletion phenotype to that of the bloodless and avascular mutant *cloche* (Dhakal et al., 2015), suggests that much of the vessel requirement for retinal development revealed by *cloche*, is important over the ∼48 hpf to 72 hpf timeframe. This knowledge of timing helps to narrow down the range of candidate factors that may be derived from endothelial cells and/or the circulation, as well as the range of candidate targets within the retina for the presumed factors. This knowledge will also help guide future studies aimed at identifying the necessary factors and their targets. It is noteworthy, however, that patterns of expression of *pax6a*, *neurod1*, and *crx*, were all disrupted in embryos depleted of vessels from ∼48 hpf to 72 hpf, but this disruption was not as severe as in *cloche* (Dhakal et al., 2015). The presence of vessels prior to 48 hpf therefore cannot be entirely discounted as also important for retinal neurogenesis. In addition, *cloche* mutant retinas display a more profound cell death phenotype than retinas of endothelial cell-depleted embryos (Dhakal et al., 2015) suggesting that earlier vessel depletion may result in greater retinal cell death. Alternatively or in addition, *cloche* may show more apoptotic profiles due to reduced clearance of dying cells because of insufficient numbers of microglia (Blume et al., 2020).

Several features of the retinal phenotype of vascular endothelial cell-depleted embryos were not phenocopied, or were not fully phenocopied, by the *sih* mutant, which specifically lacks blood flow. These features primarily included laminar disorganization and cell death. Therefore, it is likely that the requirement for vessels in promoting laminar patterning and cell survival constitutes a requirement for factors derived from endothelial cells, rather than from the circulation. Such factors may consist of paracrine signals, or may be cell contact mediated. Importantly, at this point we cannot exclude a potentially greater contribution of vascular endothelial cells to retinal development, because without circulation, endothelial cell physiology is affected. How endothelial cell physiology in turn influences the contributions of the endothelial cells to retinal development remains to be studied. Finally, although not a focus of the present work, it is important to recognize that the models for vascular disruption including *cloche−/−*, endothelial cell-depleted, and *sih−/−*, displayed defects in lens development (Goishi et al., 2006; Dhakal et al., 2015; Posner et al., 2019) (current study). Therefore, it remains possible that some of the retinal phenotypes of these vascular disruption models are secondary effects mediated by defects within the lens.

### Circulating Factors Other Than Cells of the Erythroid Lineage Are Needed to Support Retinal Cell Differentiation

The features of the phenotype of vascular endothelial-depleted embryos, which were at least in part phenocopied by *sih* mutants, included reduced proliferation, reduced photoreceptor and Müller glial differentiation, and reduced (but still well-organized) plexiform layers. It is likely that these defects contribute to the reduced overall eye growth also observed in *sih−/−*. Retinal cell proliferation and cell differentiation therefore likely each require factors derived from the circulation in order to proceed normally. The factors are not related to cells of the erythroid lineage however, since *vlt−/−* embryos displayed normal eye growth and retinal cell differentiation, nor are they related to microglia, which populate the retina in normal numbers in *sih* mutants.

Normal blood flow to the developing eye brings a number of acellular components including peptide and small molecule endocrine hormones, precursors to paracrine hormones, cholesterol, and nutrients. Several peptide signals are known to be important for the development and patterning of photoreceptors and other retinal neurons (Stenkamp, 2015; Seritrakul and Gross, 2019), including Gdf6a (Valdivia et al., 2016; DuVal and Allison, 2018), Shh (Neumann and Nuesslein-Volhard, 2000; Stenkamp et al., 2000), Fgfs (Vinothkumar et al., 2008), and Wnts (Meyers et al., 2012); however, these peptides are generally synthesized within tissues of the eye such as the lens, retinal pigmented epithelium, or the retina itself. It remains possible that a systemic peptide factor(s) is also important, however. The endocrine hormone thyroid hormone (TH) has many known roles in eye and particularly photoreceptor development (DuVal and Allison, 2018; Mackin et al., 2019; Vancamp et al., 2019), but a loss-of-function approach in zebrafish (eliminating the thyroid gland) disrupted primarily the differential expression of tandemly-replicated cone opsin genes (Mackin et al., 2019), suggesting that TH is likely not the required factor from the circulation in *sih−/−*. Retinol, a precursor for the synthesis of the paracrine signal retinoic acid (RA), can also be considered a candidate factor. RA is well-studied as a signal that promotes photoreceptor (particularly rod) differentiation (Kelley et al., 1994; Hyatt et al., 1996; Prabhudesai et al., 2005; Stevens et al., 2011; Mitchell et al., 2015), and loss-of-function approaches that impair retinol synthesis or transport result in reduced photoreceptor differentiation (Amengual et al., 2014; Shi et al., 2017). While microphthalmic, these models appear to have normal plexiform layers and do not fully phenocopy the *sih* mutants. Cholesterol is also known to be important for photoreceptor maturation and function; defects in cholesterol synthesis in humans result in Smith-Lemli-Opitz Syndrome (SLOS) (Fliesler and Xu, 2018). SLOS affects primarily rod function and causes a photoreceptor degeneration in rat models. Finally, the role of nutrients in general for retinal development has been tested in both *Xenopus* and zebrafish, through the removal of the yolk sac in order to achieve nutrient deprivation (ND) (Love et al., 2014). The outcome of ND was most evident within the CMZ, while retinal cells already committed to a particular fate continued to differentiate normally – a phenotype quite distinct from that of *sih−/−*. Therefore, the most immediately promising candidates for the factor(s) needed from the circulation consist of retinol, and/or a circulating peptide signal(s), and/or cholesterol, and/or factor(s) not considered in this discussion.

### Conclusion

The present work highlights an understudied developmental concept, that the cardiovascular system is developmentally important for more than gas exchange (Takada et al., 2017). Using genetic tools available in the zebrafish embryo, in which diffusion is a sufficient mechanism for gas exchange (Pelster and Burggren, 1996), we have identified distinctive roles for vascular endothelial cells and for circulating factors, in regulation or support of retinal development. The next steps toward identification of the factors and their targets in developing retina will involve a combination of evaluation of candidate factors, and unbiased approaches toward identifying factors and their targets.

## Data Availability Statement

The original contributions presented in the study are included in the article/Supplementary Material, further inquiries can be directed to the corresponding author/s.

## Ethics Statement

The animal study was reviewed and approved by Institutional Animal Care and Use Committees of the University of Idaho, and of Hebrew University of Jerusalem.

## Author Contributions

SD, AI, and DS conceived the project and wrote the manuscript. All authors performed experiments and analyzed the data.

## Conflict of Interest

The authors declare that the research was conducted in the absence of any commercial or financial relationships that could be construed as a potential conflict of interest.
