# Supplementary material for: Selective Requirements for Vascular Endothelial Cells and Circulating Factors in the Regulation of Retinal Neurogenesis
Source: Front Cell Dev Biol. 2021 Apr 8;9:628737. doi: 10.3389/fcell.2021.628737 (PMC8060465; doi:10.3389/fcell.2021.628737)

Supplementary Material for:

Selective Requirements for Vascular Endothelial Cells and Circulating Factors in the Regulation of Retinal Neurogenesis

Movie S1. Live differential interference contrast (DIC) imaging of circulation within the eye of a DMSO-treated doubly-transgenic (*cdh5:gal4; UAS:nfsB-mCherry*) embryo.

Movie S2. Live DIC imaging of circulation within the eye of a Met-treated, transgenic *cdh5:gal4* embryo.

Movie S3. Live DIC imaging showing absence of circulation within the eye of a Met-treated doubly transgenic (*cdh5:gal4; UAS:nfsB-mCherry*) embryo, indirectly indicating depletion of vascular endothelial cells.

Figure S1. Ocular vasculature of control and endothelial cell-depleted embryos. A.-D. Hyaloid and radial vessels in zebrafish embryos doubly transgenic for *cdh5:gal4* and *UAS:nfsB-mCherry*, and on the *kdrl:eGFP* and *gata1:dsRed* backgrounds, treated with DMSO (controls; **A**, **C**), or metronidazole (Met; **B**, **D**), viewed at 48 hours post-fertilization (hpf; **A**, **B**) and 72 hpf (**C**, **D**). The GFP+ dorsal, nasal, and ventral radial vessels (drv, nrv, vrv, respectively) and hyaloid vasculature (hv), and dsRed+ erythrocytes are visible in controls but almost undetectable in Met-treated embryos. mCherry signal is weak and difficult to detect in these images. Scale bar (in A, applies to all) = 50 µm. 4 control and 6 Met-treated embryos were examined for each condition. All embryos were PTU-treated to prevent pigmentation from interfering with imaging.

Supplementary Figure S1.

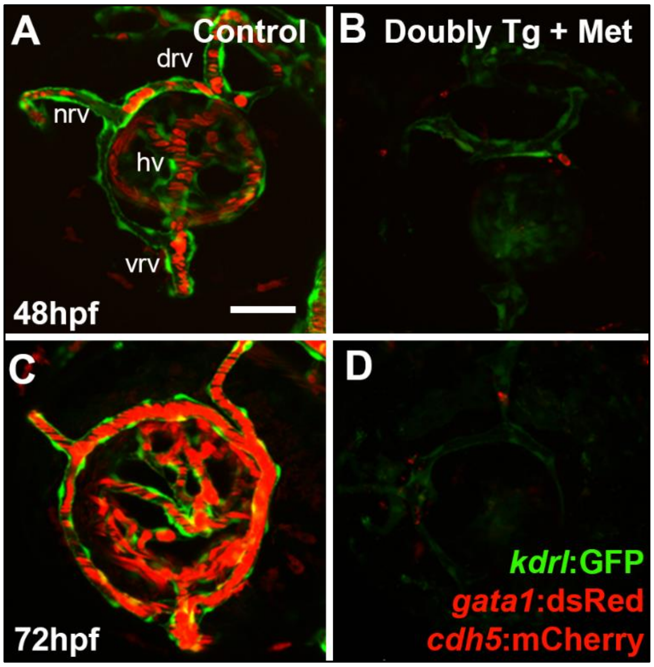

Figure S2. Retinal histology in cardiovascular disruption model systems. A.-C. Hematoxylin and eosin- (H&E) stained cryosections of doubly-transgenic (*cdh5:gal4; UAS:nfsB-mCherry*), DMSO-treated (DMSO Control, n=7; **A**); Met-treated (Endothelial Cell-Depleted, n=9; **B**); and

Met-treated clutchmates (Met Control, n=6; **C**) at 72 hpf. Control retinas show defined nuclear and plexiform layers and photoreceptor apical processes (arrow), while endothelial cell-depleted retinas are disorganized with poorly defined layers, expanded regions of eosin+ material (asterisks; *), and regions of pyknotic nuclei surround by weakly eosin+ “space” (example appears within white circular profile). **D.-E.** H&E-stained cryosections of normal clutchmates (n=10; **D**) and *sih-/-* embryos (n=9; **E**). The inner plexiform layer (IPL; brackets) is reduced in thickness in *sih-/-* compared to WT, and there is little evidence of photoreceptor apical processes (white arrow in **E**). **F.-G.** H&E-stained cryosections of normal clutchmates (n=7; **F**) and *vlt-/-* embryos (n=7; **G**). Histology of *vlt-/-* retina appears normal. ONL, outer nuclear layer; INL, inner nuclear layer; CMZ, ciliary marginal zone; GCL, ganglion cell layer. Scale bar (in B, applies to all) = 50 µm. Embryos in A-C, F-G were PTU-treated; those in D-E were not.

Supplementary Figure S2.

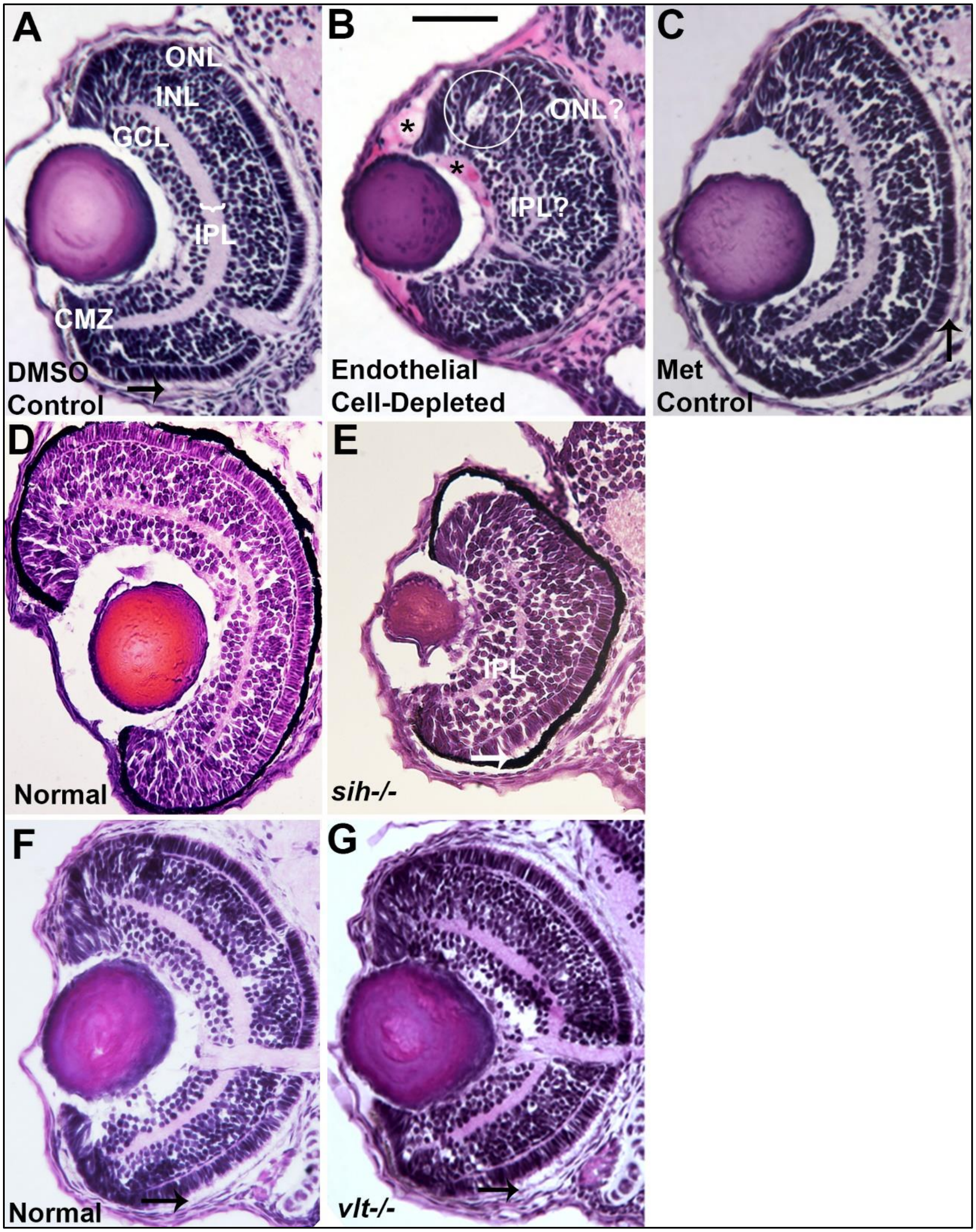

Figure S3. GFAP staining of Müller glia in cardiovascular disruption model systems. A.-C. Cryosections of doubly-transgenic (*cdh5:gal4; UAS:nfsB-mCherry*), DMSO-treated (DMSO Control, n=6; **A**); Met-treated (Endothelial Cell-Depleted, n=8; **B**); and Met-treated clutchmates (Met Control, n=7; **C**) at 72 hpf, stained with zrf1, which labels glial fibrillary acidic protein (GFAP), present within Müller glia. Control retinas show zrf1+ Müller glial processes spanning the retina with a clear radial orientation (**A**, arrow), while endothelial cell-depleted retinas display reduced zrf1 staining, processes that do not appear to span the retina, and which are not as clearly radially organized (**B**). **D.-E.** Cryosections of normal clutchmates (n=7; **D**) and *sih-/-* embryos (n=7; **E**) at 72 hpf, stained with zrf1. WT retinas show normally-patterned zrf1+ (GFAP+) Müller glial endfeet and radial processes. The *sih-/-* retinas show weak GFAP staining, primarily associated with the optic nerve head (ONH). Scale bar (in A, applies to all) = 50 µm.

Supplementary Figure S3.

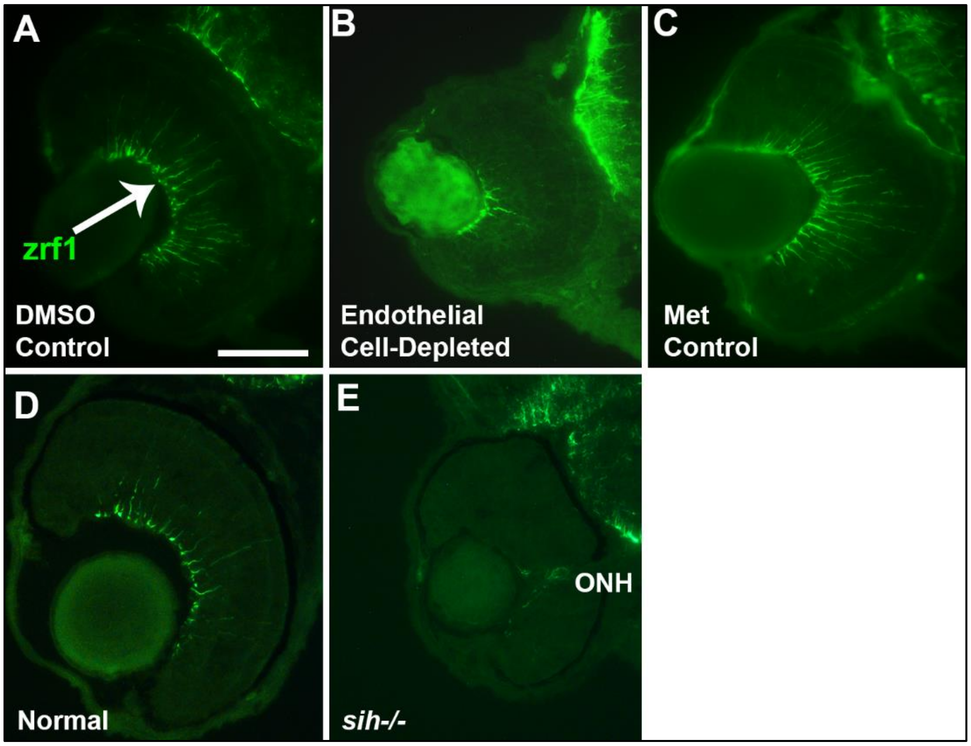

Supplement: Supplementary file 4 [file Data_Sheet_1.pdf]
